# A study on the effect of natural products against the transmission of B.1.1.529 Omicron

**DOI:** 10.1186/s12985-023-02160-6

**Published:** 2023-08-25

**Authors:** Samar Sami Alkafaas, Abanoub Mosaad Abdallah, Aya Misbah Hussien, Heba Bedair, Mahmoud Abdo, Soumya Ghosh, Sara Samy Elkafas, Wilgince Apollon, Morteza Saki, Samah A. Loutfy, Helen Onyeaka, Mohamed Hessien

**Affiliations:** 1 Molecular Cell Biology Unit, Division of Biochemistry, Department of Chemistry, Faculty of Science, Tanta University, Tanta, 31527 Egypt; 2 Narcotic Research Department, National Center for Social and Criminological Research (NCSCR), Giza, 11561 Egypt; 3 Biotechnology Department at Institute of Graduate Studies and Research, Alexandria University, Alexandria, Egypt; 4 Botany Department, Faculty of Science, Tanta University, Tanta, Egypt; 5 Department of Genetics, Faculty of Natural and Agricultural Sciences, University of the Free State, Bloemfontein, 9301 South Africa; 6 Production Engineering and Mechanical Design Department, Faculty of Engineering, Menofia University, Menofia, Egypt; 7 Department of Agricultural and Food Engineering, Faculty of Agronomy, Universidad Autónoma de Nuevo León, Francisco Villa S/N, Ex-Hacienda El Canadá, 66050 General Escobedo, Nuevo León Mexico; 8 Department of Microbiology, Faculty of Medicine, Ahvaz Jundishapur University of Medical Sciences, Ahvaz, Iran; 9 Virology and Immunology Unit, Cancer Biology Department, National Cancer Institute, Cairo University, Cairo, Egypt; 10 Nanotechnology Research Center, British University, Cairo, Egypt; 11 School of Chemical Engineering, University of Birmingham, Edgbaston, Birmingham, B15 2TT UK; 12 Division of Biochemistry, Department of Chemistry, Faculty of Science, Tanta University, Tanta, 31527, Egypt

**Keywords:** COVID-19, Omicron, Natural product, In silico, Vaccine, Molecular docking

## Abstract

**Background:**

The recent outbreak of the Coronavirus pandemic resulted in a successful vaccination program launched by the World Health Organization. However, a large population is still unvaccinated, leading to the emergence of mutated strains like alpha, beta, delta, and B.1.1.529 (Omicron). Recent reports from the World Health Organization raised concerns about the Omicron variant, which emerged in South Africa during a surge in COVID-19 cases in November 2021. Vaccines are not proven completely effective or safe against Omicron, leading to clinical trials for combating infection by the mutated virus. The absence of suitable pharmaceuticals has led scientists and clinicians to search for alternative and supplementary therapies, including dietary patterns, to reduce the effect of mutated strains.

**Main body:**

This review analyzed Coronavirus aetiology, epidemiology, and natural products for combating Omicron. Although the literature search did not include keywords related to in silico or computational research, in silico investigations were emphasized in this study. Molecular docking was implemented to compare the interaction between natural products and Chloroquine with the ACE2 receptor protein amino acid residues of Omicron. The global Omicron infection proceeding SARS-CoV-2 vaccination was also elucidated. The docking results suggest that DGCG may bind to the ACE2 receptor three times more effectively than standard chloroquine.

**Conclusion:**

The emergence of the Omicron variant has highlighted the need for alternative therapies to reduce the impact of mutated strains. The current review suggests that natural products such as DGCG may be effective in binding to the ACE2 receptor and combating the Omicron variant, however, further research is required to validate the results of this study and explore the potential of natural products to mitigate COVID-19.

**Graphical abstract:**

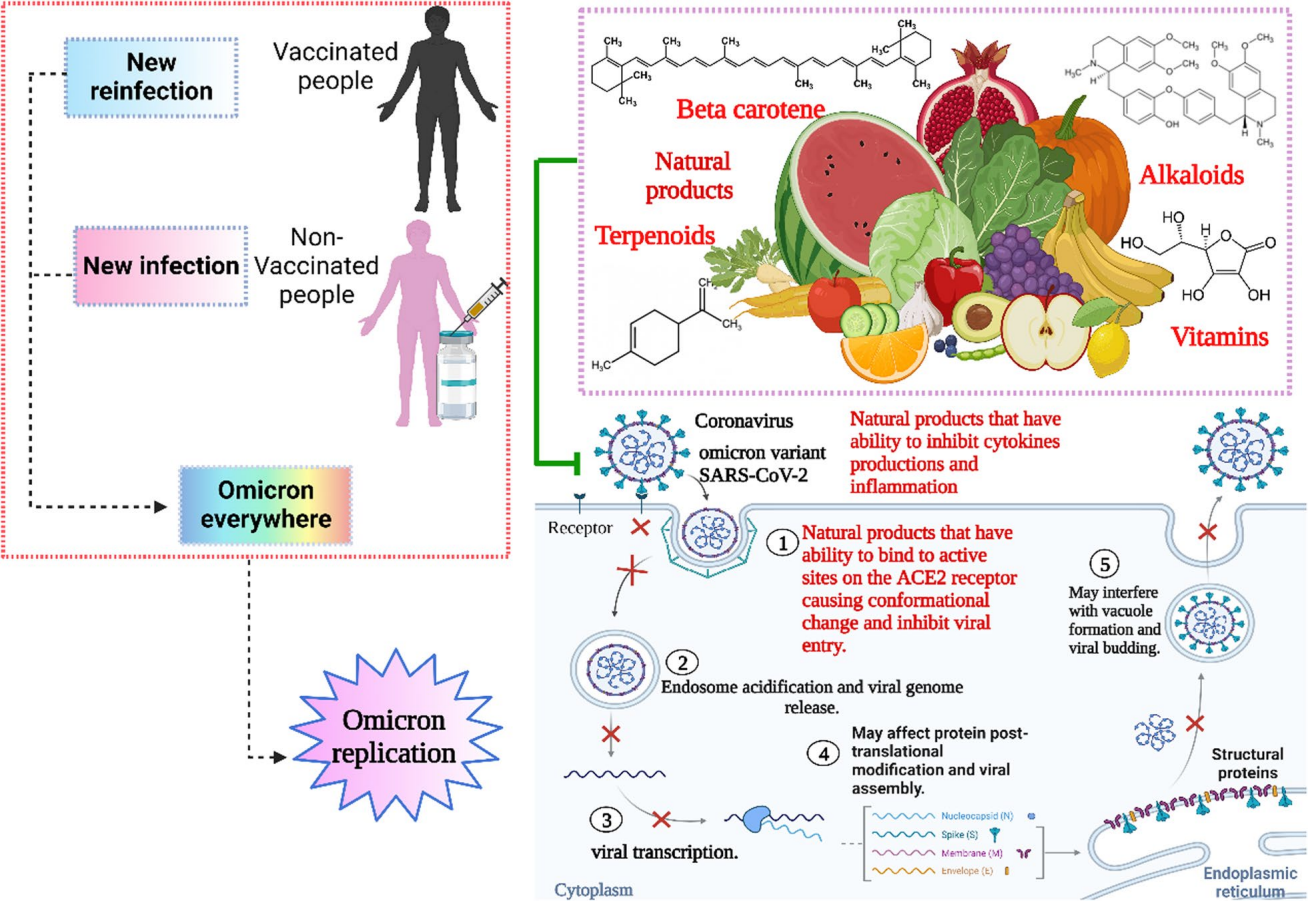

## Highlights

- There are 32 mutations in the spike of the Omicron variant.
- Non-SARS-COV-2 vaccinated individuals accelerate transmission of Omicron variant.
- Effect of mutation in transmissibility of Omicron.
- In vitro docking of natural products enhances patient immunity against Omicron virus.
- High consumption of foods rich in bioactive substances (polyphenols and vitamins) protects against Omicron infection.
- DGCG could bind to ACE2 receptor three times more effective than standard chloroquine.

## Introduction

The Omicron variant (B.1.1.529), a mutation of SARS-CoV-2, appeared in late November 2021 in South Africa [1]. According to the World Health Organization (WHO), the Omicron variant multiplies roughly 70 times faster than other variants, although with less severe infectivity [2]. The binding interaction between the spike glycoprotein of the virus and its receptor (ACE2) on cell membranes is solely responsible for Omicron internalization [3–6]. By eliminating the viral envelope, viral RNA is injected directly into the cytoplasm of the host cell [7] generating two types of RNA, ORF1a and ORF1b, which then translate into proteins pp1a and pp1ab, respectively. The translated proteins pp1a and pp1ab are proteolysed by so-called viral enzymes, for a total of 16 non-structural proteins [3]. Subsequently, the non-structural proteins form a replication/transcription complex (RNA-dependent RNA polymerase), using the genomic RNA (+) as a model. The WHO clarified that there are a high number of mutations as more than 60 substitutions, deletions, and insertions including spike containing 30 mutations [3]. These mutations increase the transmissibility of the virus in a limited period of time due to their extremely high rate of spread and ability to evade the body's immune system, despite double vaccination [8]. Our knowledge continuously increases about Omicron infectivity and mechanism of action [8]. In addition, taking strict preventive measures, such as wearing masks, social distancing, and not mixing with infected people, two doses of the vaccine were also taken to restrict the rapid Omicron infection [9]. The vaccine is the main defence against Omicron, but its effectiveness, feasibility, and safety have not been proven. Also, clinical trials are still underway, testing many drugs to reduce omicron infection. Scientists included dietary intake of specific food to prevent omicron infection or mitigate its severity [10]. It was observed that food is a crucial factor in preventing various infectious diseases [11]. For example, a high consumption of foods rich in bioactive substances [*i.e*., polyphenols and vitamins (vitamins A, C, D, E)] helps in protection of many diseases [10, 12]. Koch [13] clarified that food polyphenols have a large impact on communicable and non-communicable diseases.

In a previous study, Omrani and his co-workers [14] observed that plants with medicinal properties might be implicated in mitigation of viral invasion either via direct or indirect modulation of ACE2 activity to ameliorate COVID-19. Selected ethno-medicinal plants containing bioactive compounds which may prevent and mitigate the fusion and entry of the SARS-CoV-2 by modulating ACE2 of the host cell. Chapman and Andurkar [15] demonstrated the potential effect of polyphenols as therapeutic natural products against SARS-CoV-2. Poly phenols including glycyrrhizin, flavonoids such as quercetin, kaempferol and baicalein, and other polyphenols are the most common constituents found in Traditional Chinese Medicines that modulate inflammation and cell signaling pathways and bind viral targets demonstrating valuable effects against SARS-CoV-2 [15]. Musarra-Pizzo and co-workers [16] showed the antiviral activity exerted by natural products against human viruses. Flavonoids including anthocyanidins, flavones, flavonols, flavanones, flavan, isoflavanoids, and abiflavanoids exert antiviral effect against HSV-1, and HSV-2 entrance and infection [16]. Al-Harrasi, and co-workers [17] targeted natural products against SARS-CoV-2. Natural products, secondary metabolites show positive leads with antiviral and immunotherapy treatments using genomic studies in silico docking [17]. Al-Harrasi, and co-workers [17] evaluate the antiviral effect of the NT-VRL-1 unique terpene on Human coronavirus (HCoV-229E). They observed that natural products including Pedunculagin, tercatan, and castalin can interact strongly with SARS-receptor Cov-2’s binding site and catalytic dyad (Cys145 and His41).

Furthermore, food polyphenols aid and improve the body's defences against oxidative stress and have anti-inflammatory, antiviral, and antibacterial properties, as well as preventing cardiovascular disease, atherosclerosis, and cancer [12]. Natural products such as flavonoids, terpenoids, alkaloids, glycosides, carotenoids, phenolic and polyphenolic compounds can bind with the ACE-2 interface, causing distribution of the interaction of the omicron variant with the host ACE-2 [18].

Natural products not only disturb the binding interaction with the virus but protects against venous thromboembolism, which is a serious lung complication of Corona disease [19]. Natural products may be more important antivirals than chemical antivirals because they maintain cardiovascular health and respiratory systems, making the Omicron infected person less susceptible towards severe complications [20]. The current review, that has its base in the literature redeemed from google scholar, Publisher Medline, and an array of recent works, focused on the antiviral, antioxidant, and anti-inflammatory effects of flavonoids, terpenoids, alkaloids, glycosides, miscellaneous compounds, carotenoids, phenolic and polyphenolic compounds, which overlap with ACE-2 interface and disrupt the interaction of ACE-2 with RBD of Omicron. They act as potential natural medicines against the coronavirus family including Omicron.

## Methodology

### Study design

It is a review of relevant and current literature on the effect of natural products against the transmission of B.1.1.529 Omicron.

### Place and duration of the study

The study was conducted at the Tanta university, Egypt from February to July, 2022.

### Data collection

The data were retrieved from PubMed, Scopus, Google Scholar, and Web of Science studying literature review, old and recent textbooks, websites proceedings, and research articles. Specific keywords used were “variant of SARS-CoV-2”, “Genomic sequence”, “Mechanism of infectivity”, “ACE2 metallopeptidase domain”, “vaccinated people”, “Mutations”, “vaccination rates”, “antiviral agents”, “monoclonal antibodies”, “Antioxidant vitamins”, “Terpenoids”, “Flavonoids”, “Alkaloids”, “Carotenoids”, “Vitamins”, “In vitro docking”, and “Glycosides”. Following the outlined searches, articles were chosen based on their relevance to the objective of this review. The articles providing information are in clear relation to polyphenols and their pharmacological effects, with clear indication of their action mechanisms. Inclusion criteria of this study was set for the published articles (208 were chosen) in the last 5 years. Furthermore, molecular docking experiments with 68 natural product molecules were performed to observe their therapeutic potential against SARS-coV-2 variants.

## Omicron is a variant of SARS-CoV-2

At the end of 2019 the world focused on the COVID-19 outbreak, which resulted in more than 17 million deaths worldwide [21]. This resulted in the necessity to discover and develop safe antiviral vaccines to overcome the pandemic that threatened the stability of the world [22]. All viruses, including SARS-CoV-2, are RNA viruses that can mutate to resist vaccines [23]. The constant occurrence of mutations, led to a change in the genetic material of the strains that spread throughout the COVID-19 pandemic, which in turn led to vaccine resistance [24]. During the pandemic, numerous variant strains emerged, including Alpha, Beta, Gamma, and Delta, from the number of substitutions in the SARS-CoV-2 spike N-terminal domain (NTD) and receptor-binding domain (RBD) [25]. The WHO discovered the appearance of another variant strain of the SARS-CoV-2 virus in South Africa in late November 2021, called SARS-CoV-2 VOC, B.1.1.529 or Omicron variant, as shown in [24–26]. The WHO determined that Omicron appeared as a result of more than 30 mutations as substitutions, deletions, or insertions in the original genome of the SARS-CoV-2 [26]. These large-scale mutations affect virus characteristics including transmissibility from one patient to another, COVID-19 severity, immune escape, available diagnostics, vaccine escape and therapeutic monoclonal antibodies [23, 26]. The WHO clarified that unfortunately, Omicron variant is capable of infecting a larger number of people in less time compared to the Alpha, Beta, and Delta variants, and increases the chances of re-infection in people who have had COVID-19 before [27]. According to diagnostic reports, one of the three target genes remains undetected after PCR test and sequencing confirmation, known as S gene target failure, and should be utilized as a sign of infection by Omicron [28, 29]. A patient who may have Omicron undergoes firstly a test called Nucleic Acid Amplification Test (NAAT) or Rapid Antigen Detection Test (RADT) to detect SARS-CoV-2 infection and secondly undergoes S-gene target failure assay or another PCR-based Single Nucleotide Polymorphism (SNP) assay to confirm infection of the Omicron variant [29, 30]. Currently, reports show that the rate of Omicron infection is quicker than infection by other variants [30].

Epidemiological studies are ongoing to monitor the prevalence of infection, the severity of infection and symptoms, as well as the performance of existing vaccines against Omicron [31]. Epidemiological reports show that the symptoms of the Omicron variant include fatigue, fever, night sweats, scratchy throat, dry cough, body pain, and pain in the upper respiratory system [32, 33]. Additionally, there is no evidence of loss of taste or smell or high fever like previous variants of SARS-CoV-2 [26]. The WHO advises the need to wear face masks, maintain social distancing, and increasing the proportion of individuals receiving the vaccine [26, 34]. It clarifies that when virus maintenance of prevention measures, helps to reduce the emergence of new variants of SARS-CoV-2 [31]. Refusal to receive the vaccine for non-health reasons, mixing with infected individuals, and not adhering to measures to prevent the virus from entering the body, are the reasons for the continued existence of the pandemic [31].

## Genomic sequence of Omicron variant

**Fig. 1:**
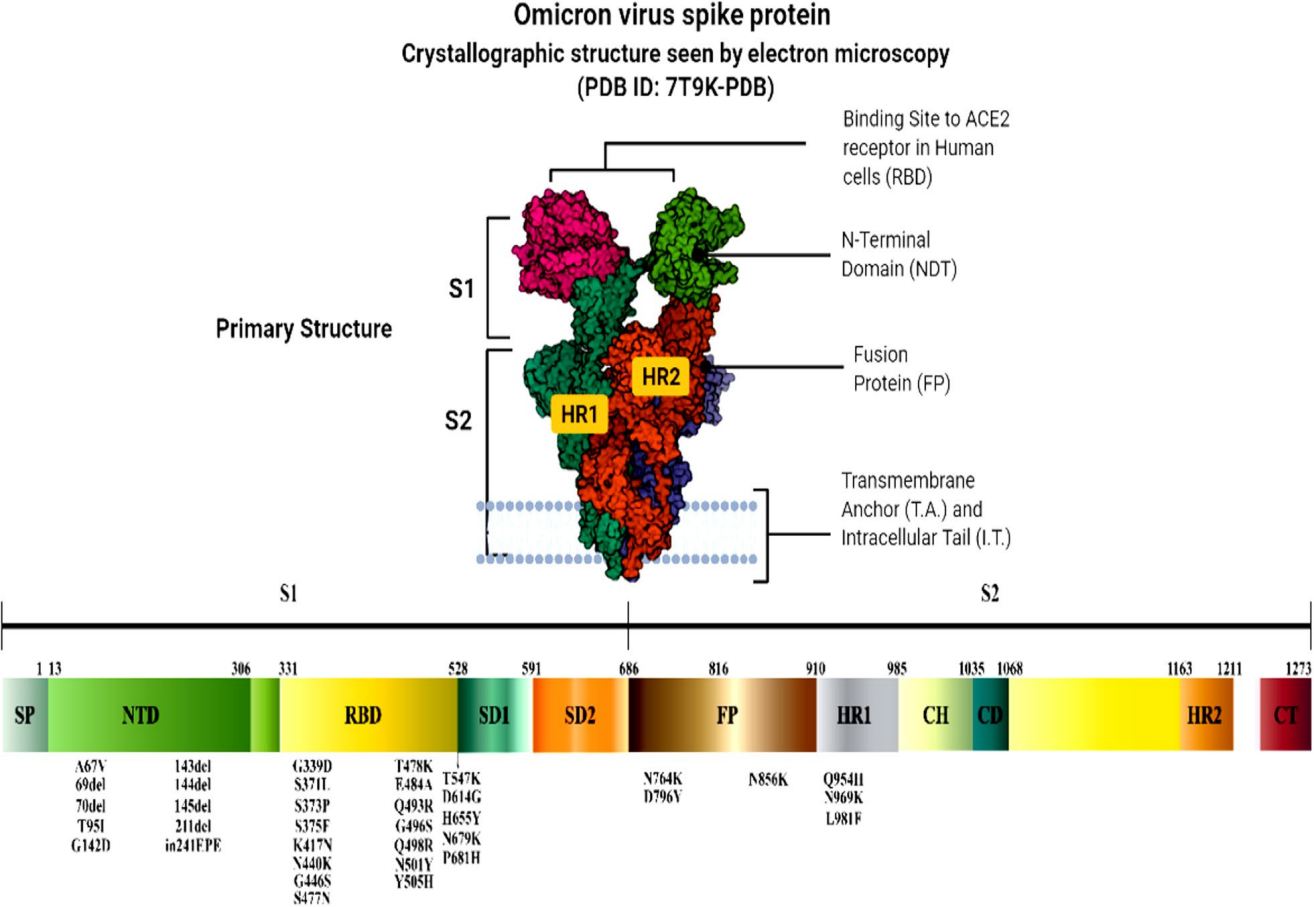
Genomic sequence of Omicron variant of PDB https://doi.org/10.2210/pdb7T9K/pdb

Analysis of the new variant Omicron revealed that there are more than 60 substitutions, deletions, and insertions including the spike protein, which contains 30 mutations [24, 35]. Omicron has six substitutions (K856R, L2084I, A2710T, T3255I, P3395H, and I3758V) within ORF1a, and two amino acid deletions, as shown in Fig. 1, in total four amino acids [31]. Additionally, Omicron comprises two substitutions, P314L and I1566V within ORF1b [31, 35]. It has been observed that ORF9b has P10S substitution and three-residue deletions at positions 27–29 [31, 35]. These large number of mutations lead to an increased rate of viral transmissibility, immune escape, and disease severity [31, 35].

There are multiple structural protein alterations, including one substitution (T9I) in the Omicron variant's envelope (E) and three substitutions (D3G, Q19E, and A63T) in the membrane (M) [31, 35]. Omicron's gene sequencing revealed that more than half of all mutations accumulate in the spike [31, 35]. There are 30 substitutions of A67V, T95I, Y145D, L212I, G339D*, S371L, S373P, S375F, K417N*, N440K^**+**^, G446S, S477N, T478K^**+**^, E484A^**+**^, Q493R^**+**^, G496S, Q498R^**+**^, N501Y, Y505H^**+**^, T547K^**+**^, D614G^**+**^, H655Y*, N679K^**+**^, P681H^**+**^, N764K^**+**^, D796Y^**+**^, N856K^**+**^, Q954H^**+**^, N969K^**+**^, and L981F, three deletions of H69/V70, G142*/V143/Y144, and N211 [31, 35]. Where the signs * and ^+^ indicate add one negative charge and add one positive charge, respectively [31]. The positive charge of the Omicron variant is increased by + 9 in the altered amino acid sequence compared to COVID-19 wild type [31, 35]. These mutations enhance the variant's transmissibility via improving the binding between spike and angiotensin-converting enzyme 2 (ACE2) [31, 35]. The transmissibility is increased further when the substitution mutations are paired with the H69/V70 deletion [31, 35]. There is one insertion of three amino acids (EPE) at position 214, where the alterations are characterized as the V143/Y144/Y145 deletion in combination with G142D and the L212 deletion in association with N211I [31, 35]. In addition, there are two mutations near the furin cleavage site, N679K and P681H mutations [31, 35]. As result, the incorporation of basic amino acids near the furin cleavage site enhances the fusion and virus infection after facilitating the cleavage of the spike into S1 and S2 [31]. This mutation enhances the infectivity of the variant [31, 35]. Electrostatic potential mapping of the wild type RBD revealed that the cationic patch is located at a distance from the receptor-binding motif (RBM) [31, 35]. After analysing the Omicron RBD, the variant revealed that a new cationic patch is produced directly on the RBM in addition to the cationic routes of the original variety, resulting in the occurrence of three nearby charged alterations (Q493R, Q498R, and Y501H) [31, 35]. Scientists predicted that the new variation Omicron would connect to the negatively charged HSPGs on the host cell surface more strongly than the original form [31]. Characterisation of several antigenic sites in RBD showed RBS-A, RBS-B, RBS-C, CR302, and S309 sites [31, 35]. The RBD of the Omicron variant spike harbours 15 substitutions, which enables the escape from antibiotic neutralization and triggers monoclonal antibiotic resistance targeting these sites [31, 35]. Omicron has two mutations, E484A and K417N, which enables escape from antibiotic neutralization, including LY-CoV555 and LY-CoV016 antibiotics respectively [31, 35]. Other alterations in the Omicron spike include D614G, N501Y, K417N, P681H, and E484 residue substitution [31, 35]. These spike mutations enhance transmissibility, virus pathogenicity and diminishes the ability of monoclonal antibody neutralization and immune evasion [31, 35].

## Mechanism of Omicron infectivity

**Fig. 2:**
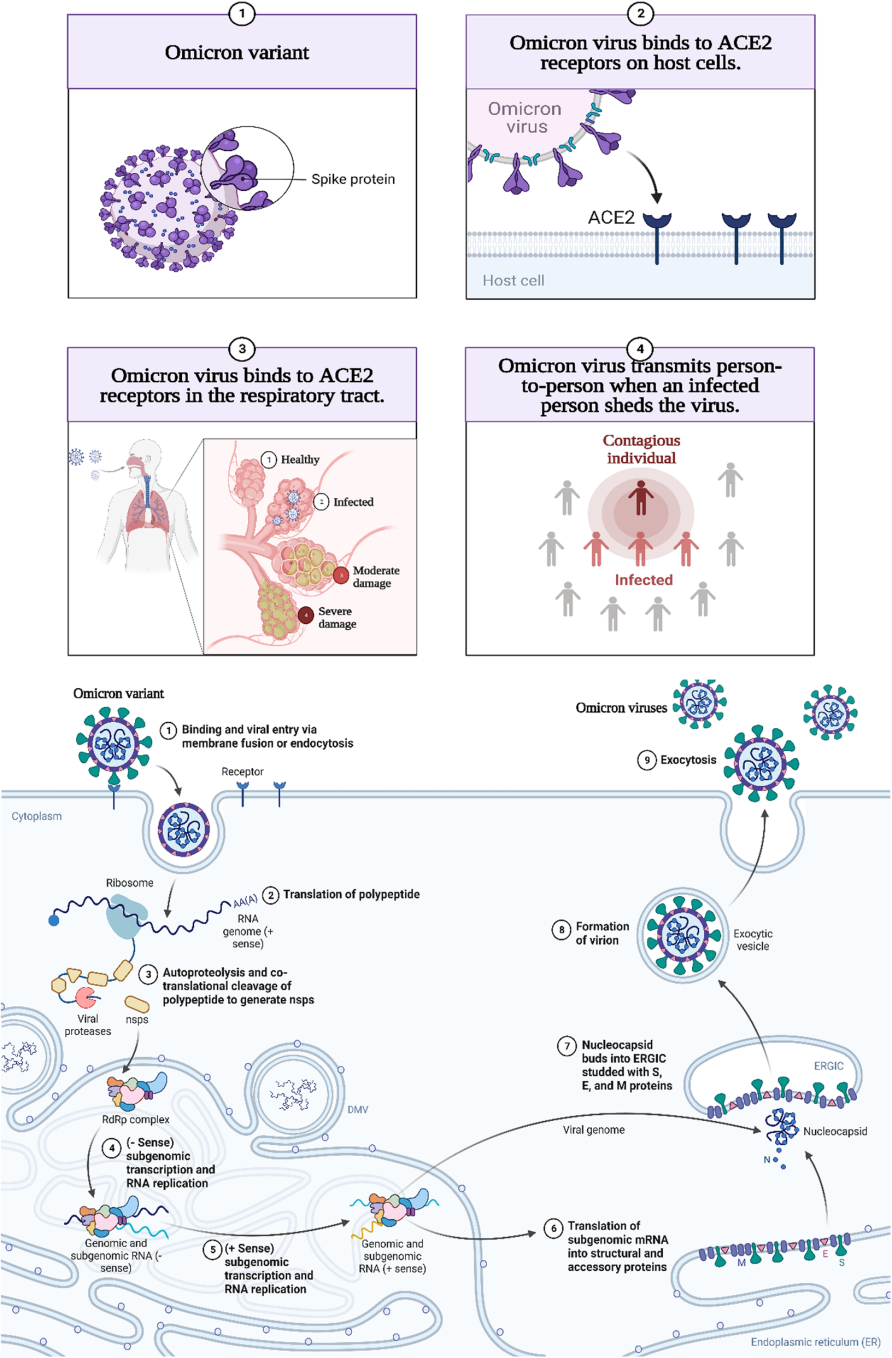
The mechanism of Omicron infectivity

According to Chen et al*.* and Walls et al*.* [36, 37], the distribution of Omicron differs depending on different viral proteins such as NSP3, NSP4, NSP5, NSP6, NSP12, NSP14, S protein, envelope protein, membrane protein, and nucleocapsid protein. The S protein receptor-binding domain (RBD) has been studied for infectivity and resistance to antibodies resulting from this new variant. Because of RBD mutations on the viral S protein, the affinity of viral S protein to the host angiotensin-converting enzyme 2, (ACE2) increases and resulting in enhanced viral infectivity. The binding of viral S-ACE2 host receptor enables the virus to enter the host cell by endocytosis, as shown in Fig. 2. According to recent research, the binding free energy (BFE) between the S-RBD and ACE2 is directly related to the infectivity of the virus. When an antibody attaches tightly to the RBD, the virus is directly internalized. Interestingly, many RBD binding antibodies are created in response to an infection or vaccination. To combat viral infections, monoclonal antibodies (mAbs) targeting the S-protein, specifically the RBD, are developed. As a result, any change in the S-protein RBD will have a significant impact on the efficacy of existing vaccines, mAbs, and the risk of reinfection [37]. The binding affinity of the receptor ACE2, RBD complex, and the cleavage site furin could dictate omicron infectivity. Naturally, the virus accelerates its evolution at the RBD, whether through changes to increase ACE2-RBD binding affinity or to avoid antibody protection. Omicron variant has three mutations at the furin site and fifteen mutations on the RBD. An efficient effective infection pathway would be when the virus has multiple RBD mutations, enhancing its infectivity, which happens with Omicron.

## Non-vaccinated individuals accelerate Omicron transmission

**Fig. 3:**
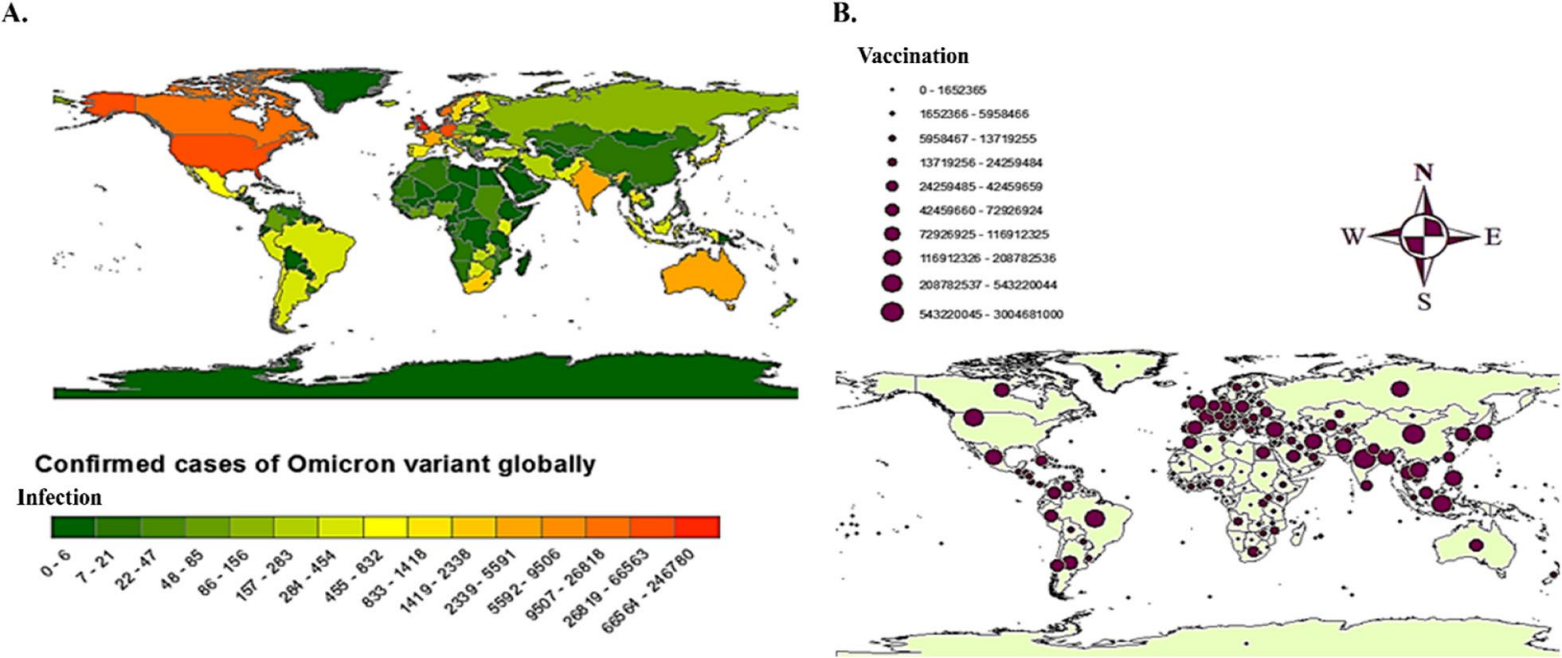
**A** Depicting the confirmed cases of Omicron variant globally. The rectangular bar chart indicates minimum (extreme left bar with dark green shade) and maximum (extreme right bar with dark red shade) infected cases with the range given against each of the bars. **B** Showing the number of vaccinated people worldwide with a range indicating the number of vaccinations from minimal (smallest circle) to maximum (largest circle). The maps have been created by using ARCGIS (https://www.esri.com/en-us/arcgis/products/arcgis-for-office/download)

**Fig. 4:**
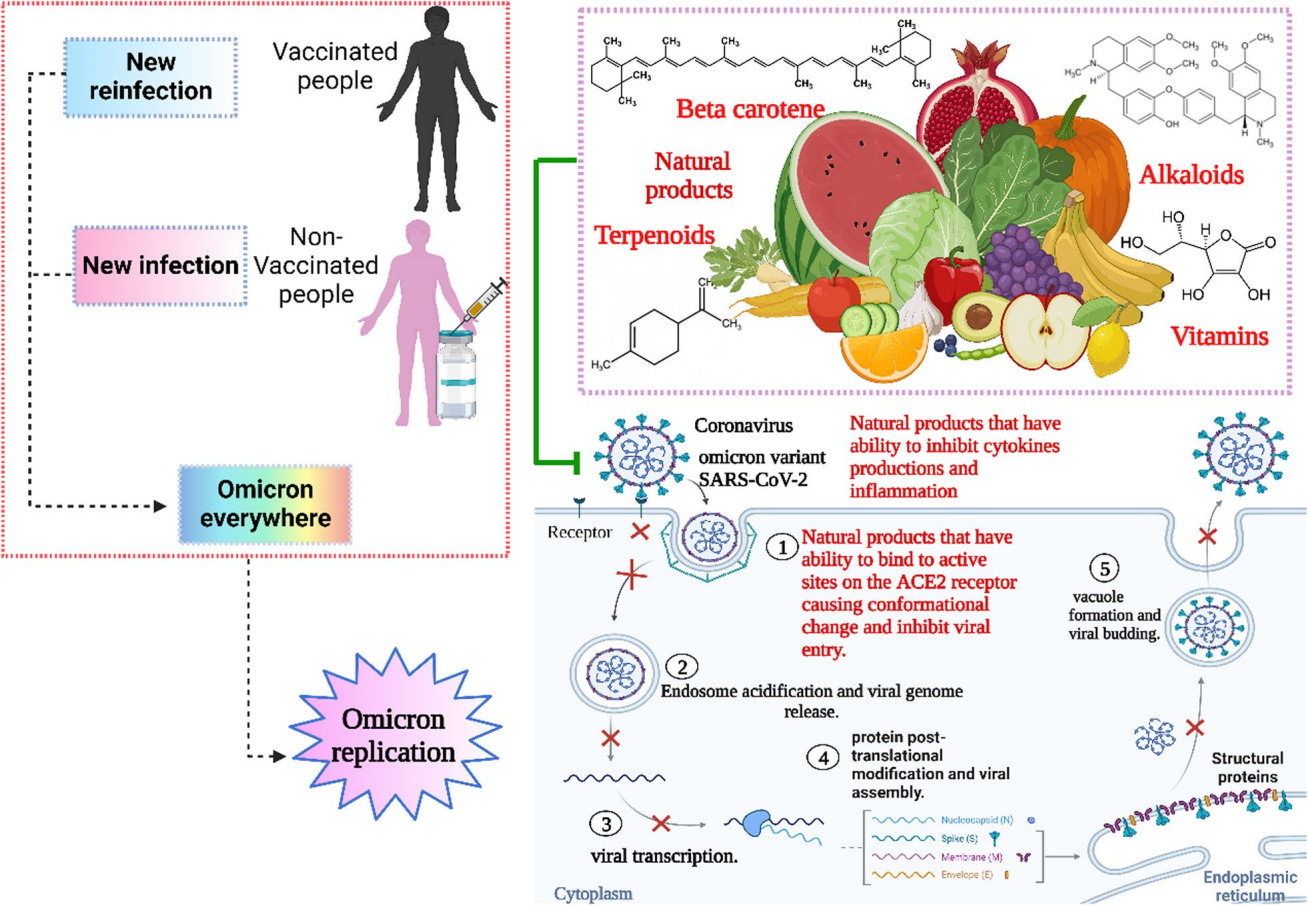
Effect of natural transmission of omicron virus between vaccinated and non-vaccinated person

The SARS-COV-2 virus is constantly changing genetically due to DNA mutations in the spike, which eventually leads to the emergence of new variants such as Alpha, Beta, Delta, and Omicron [38–41]. All new mutated genetic variants are tracked by the WHO and the Center for Disease Control [42]. In accordance with current analysis (Figure 4), Omicron infection is more prevalent in South Africa, Botswana, and Zambia, followed by most of the countries in South and Middle America, India, Australia, Northern America and Canada (Figure 3A). Notably, vaccination programmes were widely launched in most of the countries in Europe, Asia, Australia and Northern, Middle and Southern America compared to African countries (Figure 3B). These observations revealed that although there were vaccinations, the rate of infections was higher, indicating that the vaccinations was not the only solution to eradicate the pandemic. According to the WHO, Omicron could infect vaccinated persons as well because mutations in the spike protein allow it to evade the immune system and vaccine. However, it spreads more among the unvaccinated, which may contribute to the emergence of new strains.

## Effect of mutation in transmissibility of Omicron

First reports from South Africa shows that the Omicron variant is highly infective and spreads easily. The rate of Omicron infection increased dramatically and was noted around the world within a few days [43]. Rates of infection with Omicron are four times higher than the wild type SARS-CoV-2 and twice as high as the Delta variant [44]. Notably, Omicron displays effective ACE2‐mediated infection compared to the wild type SARS-CoV-2 or other variants [45, 46]. Omicron is characterized by high transmissibility due to many factors. Omicron sequencing shows that the spike protein has more than 30 mutations than the wild variant [45]. These spike mutations enable Omicron to evade immune responses and with increased transmission [45, 46]. For instance, the N501Y spike mutation enhances the binding affinity with the ACE2 receptor [27] and thus increases Omicron transmission [47]. The influence of a combination of N501Y spike mutation with S371L, S373P, S375F, Q498R, and T478Ks mutations makes the binding affinity to host receptor ACE2 stronger and enables Omicron to access the host cell easily [48–50]. The mutations H655Y and N679K that are found in the vicinity to the furin cleavage site (FCS), makes Omicron highly contagious [49]. Omicron can re-infect previously infected patients because of new mutation occurring in H655Y and N679K in the spike, which improves cleavage of the Omicron spike [51, 52]. Gene sequencing shows that P681H mutation increases the spike protein cleavage that enables Omicron to increase its transmissibility compared to wild type SARS-COV-2 [51]. The S gene target failure provides a negative outcome in PCR tests, which also permits unintended spread of Omicron [52]. Raising the electrostatic potential positive charge by + 9 in the changed amino acid of the RBD interface improves the interaction between viral spike and ACE-2, and subsequent transmissibility [53, 54]. Thus, it is important to study the genetic sequence mutation of Omicron to understand how these mutations affect viral infectivity and transmissibility [47]. These reports enable specific and precise targets to mitigate Omicron outcomes.

## Effect of Omicron on vaccinated and non-vaccinated patients

According to the WHO, B.1.1.529 variant was assigned the name Omicron on the 26^th^ of November 2021. Since then health facilities around the world have studied its behaviour and infectivity [2]. Although Omicron infectivity is less severe than other mutated variants, it is not considered a mild infection. The WHO stated that several countries mentioned that infection-severity from Omicron was less than Delta infection [55]. The low rate of severity is conferred to the high vaccination rates in such countries, so without the vaccination more people would have been hospitalised. However, the WHO stated that it is still not the appropriate time to declare the impact vaccinations [56].

Khan et al. studied 23 Omicron infected African participants for a median of 5 days after onset of symptoms and a follow-up after a median of 23 days [57]. The study resulted in a geometric mean titre (GMT) where FRNT50 increased by 13.7-fold in ten vaccinated participants with Pfizer BNT162b2 or Johnson and Johnson Ad26.CoV2. S after Omicron neutralization. Unvaccinated participants had identical Omicron neutralizing at baseline but rose from 26 to 113 (4.4-fold) at follow-up. By comparing Omicron neutralization with Delta virus neutralization, it was observed that the geometric mean titre increased by 6.1-fold from 129 to 790 in vaccinated people. The geometric mean titre only increased by 2.5-fold in unvaccinated people. As a result, Delta neutralizing was 2.1-fold higher in Omicron-infected vaccinated people at follow-up compared to unvaccinated people. The study showed that the neutralization of Delta variant was 22.5-fold higher than Omicron in people who were previously infected with Delta variant. The study concluded that Omicron re-infection would be expected to be more likely than Delta in Delta infected individuals, and in Omicron infected individuals who are vaccinated, based on relative neutralization levels. This may give Omicron an advantage over Delta, which may result in decreasing Delta infections in regions with high infection frequencies and high vaccine coverage**.**

## Current SARS-CoV-2 vaccinations and Omicron variant antibody resistance

Several methods have been used to treat viruses including antiviral agents (darunavir, atazanavir, and lopinavir/ritonavir), immune-modulatory drugs, and Tocilizumab drugs (anakinra, corticosteroids, anticoagulants, therapeutic antibodies, chloroquine, and hydroxychloroquine) [58]. A greater understanding of viral pathogenesis, defensive immunity, and natural immunity have assisted in developing SARS-CoV-2 vaccine types such as Whole Virus Vaccines, Nucleic Acid Vaccines, and Subunit Vaccines [59, 60]. Surprisingly, the Omicron variant was detected in vaccinated patients in South Africa, Hong Kong, and other countries, showing that the novel variation had invaded the immune system and necessitated the administration of informed vaccinations [61]. The Pfizer-BioNTech (BNT) and Moderna’s COVID-19 mRNA vaccines, which proved to be efficient against many COVID-19 variants, were significantly less efficient against the Omicron variant [62]. Both vaccinations create high-titre anti-COVID-19 Spike (S) protein-specific antibodies that can fight the initial prevalent SARS-CoV-2 strains as well as later variants [56].

The neutralizing activity of sera from vaccinated people who showed decreased protection against Omicron is reduced by extensively mutated Omicron variant spike protein [46]. Recent research showed that only 20% and 24% of BNT162b2 receivers had neutralizing antibodies against the Omicron mutants HKU691 and HKU344R346K, respectively [61]. About 75% of Omicron variant-positive patients in a South African hospital were unvaccinated and had severe results compared to vaccinated patients. Therefore, it is expected that current COVID-19 vaccinations can protect vaccinated persons until the Omicron variant‐specific COVID‐19 vaccine is available [61].

Since SARS-CoV-2 was identified as a potential cause of COVID 19, monoclonal antibodies have been extracted, mostly from B cells of patients who recently recovered from SARS-CoV-2 infection, patients infected with severe acute respiratory syndrome coronavirus, and by immunization of humanized mice [63]. Finally, all monoclonal antibodies that neutralize SARS-CoV-2 target the surface spike glycoprotein, which binds with the angiotensin-converting enzyme 2 (ACE 2) receptor located on many cell types and promotes viral entry into host cells [64]. Since the summer of 2020, clinical trials on several monoclonal antibodies against SARS-CoV have been conducted [63, 65]. Some products include one or a combination of 2 monoclonal antibodies and have been designed to target different sites on the spike protein [63].

Even though more than 75 monoclonal antibodies have proved effective in the treatment and prevention of several infectious diseases, there are limitations to their use in the treatment of COVID-19. The first consideration is the unknown localities of bioavailability of Immunoglobulin G in the infected cells. The second is the evolution of resistant virus variants [63]. Consequently, monoclonal antibodies should be well selected to target conserved regions of the viral spike. Eli Lilly mAb is a cocktail of LY-CoV555 (Bamlanivimab) and LY-CoV016 (aka Etesevimab) and was mainly developed using the SARS-CoV-2 virus as a model. It interacts with the S protein, receptor-binding domain (RBD), and blocks COVID-19 entry to the cell [66, 67]. Omicron’s spike mutations induced changes in the mAb LY-CoV016 and reduced the complex efficacy. According to a prior study, LY-CoV555 is susceptible to the E484K mutation in Beta and Gamma variants [67]. Chen et al. [67] predicted that Eli Lilly mAb cocktail was withdrawn from the market because both LY-CoV555 and LY-CoV016 were susceptible to Omicron mutation-induced efficacy reduction.

Furthermore, another cocktail of REGN10933 and REGN10987 (aka Casirivimab and Imdevimab, respectively) was developed against COVID-19 as Regeneron mAbs [68]. They are better than Eli Lilly mAb because they bind to different areas of the RBD and do not overlap each other [67]. Although K417N and E484A Omicron mutations reduce the binding of the REGN10933-RBD complex, most other mutations strengthen the binding of the complex in an opposite manner, which in turn neutralizes the first one. Chen et al*.* expect that if Omicron does not improve the efficacy of the Regeneron cocktail, it will have a minor detrimental impact [67].

A cocktail of Celltrion's antibody CT-P59 (aka Regdanvimab, PDB ID: 7CM4) and CT-P63 were efficacious against COVID-19. However, Omicron mutations E484A, Q493R, and Q498R reduced the binding of the CT-P59-RBD complex [66]. Consequently, caution has been advised by Chen et al. [67] when using Celltrion's Regdanvimab. Almost all of Omicron's mutations have a minor effect on antibodies C135 and C144, except for S317L. In contrast, host receptor ACE2, which binds to the receptor-binding domain (RBD) and blocks COVID-19 entry to the cell, prevented Omicron spike penetration efficiently [63]. Sotrovimab is one of the potential antibodies utilized against COVID-19 due to high inhibition effect against entry of SARS-COV2 virus and related variant viruses to host cells [62, 69].

## Antioxidant vitamins and Omicron

The high affinity of Omicron to ACE2 host receptor results in decreasing its bioavailability that blocks the way for Angiotensin II to bind. Instead, the free Ang II interacts with AT1R, which leads to the activation of NADPH oxidase (NOX) [70]. This results in increased oxidative stress by the formation of ROS in a NAD (P)H-dependent mechanism [70]. Omicron patients develop a cytokine storm, which is a hyperinflammatory response marked by high levels of cytokines, such as IL-1β, IL-2, IL-6, IL-7, IL-8, IL-10, or IL-17, interferon (IFN)γ, tumour necrosis factor-alpha (TNFα) and chemokines, leading to organ failure and death. Many natural products have potential activity against the Omicron variant and curb the progression of symptoms, as shown in Table 1. The natural components include melatonin, ergothioneine, hesperidin, curcumin, and vitamins (C, D, E) [71, 72]. Furthermore, citrus fruits are high in vitamin C and hesperidin, both of which have antioxidant and anti-inflammatory activities. Hesperidin boosts the antioxidant defence system opposed to Omicron as a reactive oxygen species (ROS) scavenger, especially against superoxide and hydroxyl radicals [73]. Hesperetin, a hesperidin derivative, has anti-oxidative properties, significantly reducing nitric oxide generation by LPS-stimulated microglial cells [74]. It reduces the inflammation markers and increases cellular anti-oxidative defence through the ERK/Nrf2 signalling pathway. Hesperidin efficiently targets the binding interface of Omicron spike protein and ACE2 resulting in inhibition of viral entry by endocytosis. Endocytosis is an important process that related with endocytic proteins that act as potential targets for inhibitors [75]. The natural compound curcumin acts as an Nrf2 activator and protects patients against cytokine storm [76]. Curcumin exerts a notable effect in regulating pro- and anti-inflammatory factors such as IL-6, IL-8, IL-10, and COX-2, and acts as a ROS scavenger [76]. Curcumin reduces ROS generation through the NADPH-dependent mechanism, which is induced in Omicron infections by inhibiting NADPH oxidase activity.

**Table 1:** Natural products and their sources with their antiviral action

	Type	Source	Mode of action	References
*Flavonoids*				
1	Caflanone	*Cannabis sativa*	It has a great binding affinity towards ACE2 in the host cell instead of the spike of coronavirus glycoprotein-binding site and so it triggers inhibition of the virus entry via endocytosis It also decreases the expression of cytokines such as viz. IL-1β, IL-6, IL-8, Mip-1α, and TNF-α	[77]
2	Hesperetin	Citrus fruits such as *Cordia sebestena L* *Origanum majorana L*	It has a great binding affinity towards ACE2 in the host cell instead of the spike of coronavirus glycoprotein-binding site and so it triggers inhibition of the virus entry via endocytosis It is considered a potential antioxidant scavenger against superoxide, hydroxyl radicals, and nitric oxide production	[77–80]
3	Myricetin	*Myrica rubra* *Hypericum afrum Abelmoschus moschatus*	It has a great binding affinity towards ACE2 in the host cell instead of the spike of coronavirus glycoprotein-binding site and so it triggers inhibition of the virus entry via endocytosis	[77, 81, 82]
4	Linebacker	*Cannabis sativa*	It is considered a novel prophylactic and a therapeutic natural product. Because it has a great binding affinity towards ACE2 in the host cell instead of the spike of coronavirus glycoprotein-binding site and so it triggers inhibition of the virus entry via endocytosis	[77]
5	Pectolinarin	*Cirsium subcoriaceum* *Cirsium chanroenicum* *Cirsium setidens*	It has antiviral activity against 3Clpro which main protease that enables the processing of the first proteins transferred from the viral genome-pp1a and pp1ab-into functional proteins in the host cell during the assembly of the virus	[18, 83–85]
6	Quercetin	*Euonymus alatus* *Rosa canina* *Toona sinensis* *Allium cepa* *Malus domestica* *Brassica cretica Lam*	It interacts strongly with the main protease of coronavirus result the stopping of viral assembly	[79, 86–88]
7	Baicalin	*Scutellaria baicalensis*	It has a strong specific binder to 3Clpro which main protease that enables the processing of the first proteins transferred from the viral genome-pp1a and pp1ab-into functional proteins in the host cell during the assembly of the virus	[89–91]
8	Diosgenin	*Hellenia speciosa* *Solanum virginianum* *Dioscorea bulbifera* *Dioscorea nipponica*	It possesses the potential against the infection of the virus. It has an affinity to bind to ACE-2 in the host resulting in inhibition of viral entrance	[89, 92, 93]
9	Luteolin	Honeysuckle *Martynia annua L* *Lonicera japonica* *Colchicum ricthii* *Elsholtzia rugulosa*	It is an important flavonoid because it has potent antiviral activity against coronavirus. It has an activity to bind with ACE-2 in the host cell instead of the virus. Also, it has antiviral activity against 3Clpro” or “Mpro” and prevents the assembly of the virus	[93–96]
10	Herbacetin	*Linum usitatissimum* *Rhodiola rosea* *Ephedra sinica*	It has potent antiviral activity against coronavirus. It has antiviral activity against 3Clpro and prevents the assembly of the virus	[91, 97, 98]
11	Cyanidin	*Prunus cerasus* *Oryza sativa*	It binds to the Asp761 catalytic residue of the virus and triggers down-regulate the RNA-dependent RNA polymerase and prevents the replication of the virus	[99, 100]
12	Kaempferol	Propolis *Euonymus alatus* *Vachellia nilotica* *Persicaria tinctoria* *Eruca vesicaria* *Lagenaria siceraria* *Nelumbo nucifera*	It triggers down-regulation of TMPRSS2 which is the key receptor for the entry of the virus. And so it acts as an antivirus natural product	[101–105]
13	Rutin	*Dendropanax morbifer* *Triticum aestivum* *Chrozophora tinctoria* *Spermacoce hispida* *Calendula officinalis*	It has potent antiviral activity by high affinity to bind with Mpro and inhibits the viral assembly	[106–110]
14	Narcissoside	*Morinda citrifolia* *Polygonatum odoratum* *Lolium multiflorum*	It has a higher affinity with the protein complex 6W63 of coronavirus which is the main protease of the virus causing viral inhibition	[111–113]
15	Isorhamnetin-3-O-B-D-Glucoside (IRG)	*Calendula officinalis* *Chrysanthemum morifolium* *Salvadora persica*	It has high affinity, good stability, and flexibility with Mpro by binding resulting in inhibition of viral replication	[106, 114]
16	Procyanidin	*Uncaria tomentosa* *Malus domestica* *Hypericum perforatum* *Sclerocarya birrea* *Machaerium floribundum*	It has a potential interaction with viral protein which inhibits the function and the infection process It has a low affinity to bind to 3CLpro resulting in inhibition of viral replication	[115–118]
17	Nicotiflorin	*Caragana spinosa* *Nymphaea candida* *Zeravschania aucheri* *Edgeworthia chrysantha* *Brickellia cavanillesii*	It has potential an inhibitory effect against the virus by binding to the catalytic dyad of 3CL pro resulting in inhibition of viral replication	[107, 119–121]
18	Broussochalcone A	*Broussonetia papyrifera*	It has high affinity, higher stability, and less conformational fluctuations in the Mpro of coronavirus resulting in inhibition of viral replication	[122, 123]
19	Broussoflavan A	*Broussonetia papyrifera*	It is considered a promising natural drug against coronavirus. It interacts with Mpro resulting in the Broussoflavan A-Mpro complex which is characterized by high stability and resulting inhibition of viral replication	[122]
20	Fisetin	Pigmented fruits and vegetables *Elaeagnus indica* *Hymenaea courbaril Toxicodendron vernicifluum*	It targets the protein 3CLpro by making a hydrogen bond with the Cys145A amino acid residues of the virus resulting in inhibition of viral replication	[124–126]
21	Isolicoflavonol	*Glycyrrhiza uralensis* *Broussonetia papyrifera* *Macaranga indica* *Macaranga conifera*	It targets the 3CLpro protein and interacts by the formation of hydrogen bonds resulting in inhibition of viral assembly	[120, 127, 128]
22	Licoisoflavone B	*Lupinus albus* *Lupinus angustifolius* *Sophora moorcroftiana*	It targets the 3CLpro protein and interacts by the formation of hydrogen bonds resulting in inhibition of viral assembly	[124]
23	Limonene	Essential oils of several citrus plants	It protects lung fibrosis by interacting with PI3K and NF-κB p65 result inhibition of expression and phosphorylation	[129]
24	β-Carophyllene	*Ocimum spp* *Piper spp* *Salvia officinalis* *Cinnamomum spp* *Piper cubeb* *Humulus lupulus* *Lavandula angustifolia* *Origanum vulgare* *Rosmarinus officinalis*	It targets the 3CLpro protein and interacts by the formation of hydrogen bonds resulting in inhibition of viral assembly	[130]
*Terpenoids*				
25	Crocin	*Crocus sativus* *Gardenia jasminoides* *Laurus nobilis* *Zingiber zerumbet* *Dioscorea japonica* *Teucrium ramosissimum*	It acts as promising as terpenoid's natural product against coronavirus. It interacts with the main protease of the virus resulting in inhibition of viral assembly	[83, 131–133]
26	Sarsasapogenin	*Anemarrhena asphodeloides* *Asparagus officinalis* *Yucca glauca*	It has a potential effect against the virus by forming a strong hydrogen bond with Lys290 of Nsp15 of the virus resulting in inhibition of viral infection	[134–136]
27	Ursonic Acid	*Piper betle* *Ziziphus jujube* *Lantana camara* *Catharanthus roseus*	It has an affinity to bind to Nsp15 of the virus resulting in stable complexes which inhibit viral infection	[136–138]
28	Carvacrol	*Lippia multiflora* *Origanum acutidens* *Origanum dictamnus* *Thymus vulgaris*	It has potential activity against the virus by binding to the S protein of the virus. Also, its structure of hydroxyl with a phenyl ring enables the virus to have antiviral potency against the virus	[139–142]
29	Glycyrrhizic acid	*Glycyrrhiza glabra*	It triggers induction of interferon result prevention viral replications	[143, 144]
30	Crocin	*Crocus sativus* *Gardenia jasminoides* *Laurus nobilis* *Zingiber zerumbet* *Dioscorea japonica* *Teucrium ramosissimum*	It acts as promising as terpenoid's natural product against coronavirus. It interacts with the main protease of the virus resulting in inhibition of viral assembly	[83, 131–133]
26	Sarsasapogenin	*Anemarrhena asphodeloides* *Asparagus officinalis* *Yucca glauca*	It has a potential effect against the virus by forming a strong hydrogen bond with Lys290 of Nsp15 of the virus resulting in inhibition of viral infection	[134–136]
27	Ursonic acid	*Piper betle* *Ziziphus jujube* *Lantana camara* *Catharanthus roseus*	It has an affinity to bind to Nsp15 of the virus resulting in stable complexes which inhibit viral infection	[136–138]
28	Carvacrol	*Lippia multiflora* *Origanum acutidens* *Origanum dictamnus* *Thymus vulgaris*	It has potential activity against the virus by binding to the S protein of the virus. Also, its structure of hydroxyl with a phenyl ring enables the virus to have antiviral potency against the virus	[139–142]
29	Glycyrrhizic acid	*Glycyrrhiza glabra*	It triggers induction of interferon result prevention viral replications	[143, 144]
30	Crocin	*Crocus sativus* *Gardenia jasminoides* *Laurus nobilis* *Zingiber zerumbet* *Dioscorea japonica* *Teucrium ramosissimum*	It acts as promising as terpenoid's natural product against coronavirus. It interacts with the main protease of the virus resulting in inhibition of viral assembly	[83, 131–133]
26	Sarsasapogenin	*Anemarrhena asphodeloides* *Asparagus officinalis* *Yucca glauca*	It has a potential effect against the virus by forming a strong hydrogen bond with Lys290 of Nsp15 of the virus resulting in inhibition of viral infection	[134–136]
27	Ursonic Acid	*Piper betle* *Ziziphus jujube* *Lantana camara* *Catharanthus roseus*	It has an affinity to bind to Nsp15 of the virus resulting in stable complexes which inhibit viral infection	[136–138]
28	Carvacrol	*Lippia multiflora* *Origanum acutidens* *Origanum dictamnus* *Thymus vulgaris*	It has potential activity against the virus by binding to the S protein of the virus. Also, its structure of hydroxyl with a phenyl ring enables the virus to have antiviral potency against the virus	[139–142]
29	Glycyrrhizic Acid	*Glycyrrhiza glabra*	It triggers induction of interferon result prevention viral replications	[143, 144]
*Alkaloids*				
30	Quinadoline B	Fungus *Cladosporium sp* *Aspergillus giganteus* *Ulva lactuca* *Scedosporium apiospermum*	It has anti-viral activity against the virus by binding the sites of PLpro through H-bonds formation and interacting with the S protein of the virus. It exerts the formation of H-bonding with nsp15 and exerts an effect on the Val292 site of the virus resulting in inhibition of viral infection	[145–147]
31	Scedapin C	*Aspergillus giganteus*	It interacts with 3CLpro resulting in inhibition of its activity resulting inhibition of viral assembly	[145]
32	Berberine	*Hydrastis Canadensis* *Tinospora cordifolia* *Berberis vulgaris*	It interacts with 3CLpro resulting in inhibition of its activity resulting inhibition of viral assembly	[148–150]
33	Nigellidine	*Nigella sativa*	It interacts with the structural protein Nucleocapsid (N) protein of the virus result inhibition of viral activity. And also, it interacts with Mpro resulting in inhibition of viral replication	[151]
34	Noscapine	*Papaver somniferum*	It has a higher affinity towards the pocket-3 of Mpro by the formation of hydrogen bonds resulting in inhibition of viral replication	[136, 152, 153]
35	Oxoturkiyenine	*Hypecoum pendulum*	It interacts with cathepsin L which is the key factor in the receptor of the host cell resulting in inhibition of viral entry	[154–156]
36	3α,17α-Cinchophylline	*Cinchona calisaya*	It has antiviral activity against the virus by interacting with cathepsin L which is the key factor of the receptor of the host cell resulting in inhibition of viral entry	[154]
37	Speciophylline	*Uncaria tomentosa* *Uncaria lanosa* *Uncaria bernaysii*	It exerts a high affinity to interact with 3CLpro and prevent viral replication	[115, 157]
38	Cadambine	*Uncaria tomentosa* *Uncaria rhynchophylla* *Neonauclea purpurea*	It has a significant affinity to bind with 3CLpro and prevent viral replication	[115, 158]
*Glycosides*				
39	Delphinidin 3,3′-Di-Glucoside-5-(6-P-Coumarylglucoside) (DGCG)	*Gentiana cv. Albireo*	It has a potential function to interdict the main protease of the virus resulting prevents viral infection	[159]
40	Pelargonidin	*Pomacea maculata*	It is a derivative of anthocyanin which has potential activity against the main protease resulting in inhibition of viral assembly	[159, 160]
41	Digitoxigenin	*Nerium oleander* *Digitalis lanata*	It acts as an antiviral agent against coronavirus by interacting with the main protease of the virus resulting in inhibition of viral assembly	[83]
*Quinones*				
42	Anthraquinone	Plants *cascara buckthorn*	It interacts with non-structural polypeptides of the virus resulting prevent of viral activity	[161]
*Monolignols*				
43	Anethole	*Pimpinella anisum* *Illicium verum* *Vepris madagascarica*	It interacts with the S protein of the virus by forming hydrogen bonds resulting in inhibition of virus infectivity	[139, 162, 163]
44	Cinnamaldehyde	*Cinnamomum verum*	It interacts with S protein of the virus by forming hydrogen bonds resulting in inhibition of virus infectivity	[139, 164]
*Phenolic and Polyphenolic Compounds*				
45	Curcumin	*Curcuma longa*	It triggers reducing of inflammatory cytokines which result from infection. Also, it down-regulates nucleoprotein expression preventing infection. Furthermore, 3CLpro prevents viral replication	[165, 166]
46	Syn-16	*Anthoxanthum odoratum* *Galium odoratum* *Hierochloe odorata* *Melilotus Tonka*	It acts as coumarin derivatives that possess a potential antiviral effect against the virus by interacting with S1, S2, and S5 pocket residues of the virus. Also, it binds with 3CLpro and prevents viral replication and mutation	[73]
47	Gallocatechin gallate	*Saxifraga spinulosa* *Camellia sinensis* *Diospyros kaki*	It has potent antiviral activity against the virus by interacting with S protein of the virus	[167, 168]
48	Ararobinol	*Senna occidentalis* *Frangula caroliniana* *Senna siamea*	It has a potential affinity towards cathepsin L which is the key factor in the receptor of the host cell resulting in inhibition of viral entry	[154, 169, 170]
49	Gingerol	*Zingiber officinale* *Aframomum melegueta*	It possesses a potential antiviral effect against the virus by interacting with 3CLpro preventing viral replication and mutation	[171, 172]
50	Nat-1 (coumarin analog)	*Anthoxanthum odoratum* *Galium odoratum* *Hierochloe odorata* *Melilotus Tonka*	It possesses a potential antiviral effect against the virus by interacting with 3CLpro preventing viral replication and mutation	[73]
*Miscellaneous Compounds*				
51	Isochaetochromin D1	*Fusarium sp*	It interacts with the nonstructural protein 15 (nsp15) of the virus resulting inhibition of viral infection	[145]
52	Bisindigotin	*Isatis tinctoria* *Persicaria tinctoria*	It binds with the S protein of viral spike resulting in inhibition of viral infectivity	[164, 173]
53	Edgeworoside C	*Edgeworthia gardneri* *Edgeworthia chrysantha*	It interacts with TMPRSS2 and triggers down-regulation of its expression which is the key receptor for the entry of the virus	[154, 174]
54	Adlumidine	*Fumaria indica* *Pseudofumaria lutea* *Dactylicapnos torulosa*	It interacts with TMPRSS2 and triggers down-regulation of its expression which is the key receptor for the entry of the virus	[154, 175, 176]
55	Asparagoside-C	*Asparagus racemosus*	It has potent antiviral activity against the virus by interacting with S protein of the virus and inhibits infectivity	[177]
56	Asparagoside-D	*Asparagus racemosus*	It has potent antiviral activity against the virus by interacting with S and N proteins of the virus result inhibition of infectivity	[177]
57	Asparagoside-F	*Asparagus racemosus*	It has potent antiviral activity against the virus by interacting with the N protein of the virus and inhibits infectivity	[177]
58	3-(3-Methylbut-2-enyl)-3,4,7-trihydroxyflavane (MTHF)	*Broussonetia papyrifera*	It interacts with Mpro the main protease and makes a stable complex resulting in inhibition of virus replication	[122]
59	Kazinol F	*Broussonetia papyrifera*	It interacts with the catalytic residue of Mpro preventing viral replication and mutation	[122]
60	Kazinol J	*Broussonetia papyrifera*	It interacts with the catalytic residue of Mpro preventing viral replication and mutation	[122]
61	Cinnamyl acetate	*Cinnamomum verum* *Cinnamomum osmophloeum*	It has potent antiviral activity against the virus by interacting with the S protein of the virus and inhibits infectivity	[139, 178, 179]
62	L-4-terpineol	*Oil of tea tree and lavender* *Artemisia herba-alba* *Pistacia chinensis* *Nigella sativa*	It has potent antiviral activity against the virus by interacting with the S protein of the virus and inhibits infectivity	[139, 180, 181]
63	Allicin	*Allium sativum*	It possesses a potential antiviral effect against the virus by interacting with 3CLpro preventing viral replication and mutation	[171]
64	Lianhua qingwen	*Forsythia suspense* *Lonicera japonica* *Ephedra sinica* *Gypsum fibrosuum* *Rheum palmatum*	It has a great binding affinity towards ACE2 in the host cell instead of the spike of coronavirus glycoprotein-binding site and so it triggers inhibition of the virus entry via endocytosis	[182, 183]
65	Pudilan (PLD)	*Scutellaria baicalensis* *Corydalis bungeana*	It has a great binding affinity towards ACE2 in the host cell instead of the spike of coronavirus glycoprotein-binding site and so it triggers inhibition of the virus entry via endocytosis	[184]
66	Coronil	*Withania somnifera* *Tinospora cordifolia* *Ocimum tenuiflorum*	It reduces cytokine production after viral infection It has a great binding affinity towards ACE2 in the host cell instead of the spike of coronavirus glycoprotein-binding site and so it triggers inhibition of the virus entry via endocytosis	[185–187]
*Carotenoids*				
67	Astaxanthin	*Haematococcus pluvialis* *Chlorella zofingiensis* *Chlorococcum* *Phaffia rhodozyma*	It has potent antioxidant activity, free radical scavengers, and anti-inflammatory agents	[188, 189]
68	Polyketides Emodin	*Aspergillus spp* *Penicillium spp*	It interacts with the S protein of the spike and inhibits viral infectivity. Furthermore, it has a great binding affinity towards ACE2 in the host cell instead of the spike of coronavirus glycoprotein-binding site and so it triggers inhibition of the virus entry via endocytosis	[190]

Vitamins play an important role in controlling the production of ROS. Vitamin D plays a vital role in developing antioxidant defence and controlling systemic inflammation [71]. It helps in depleting one of the sources of ROS developed in Omicron infections by suppression of NADPH oxidase (NOX). In addition, Vitamin D induces the expression of CAT, GPx, GSR, and other antioxidant defence system molecules, which result in a reduction of oxidative stress and cellular oxidation [191]. Vitamin D reduces the expression of pro-inflammatory cytokines, which prevents immune-mediated injury in Omicron infections and reduces the severity of cytokine storm. Vitamin D suppresses the development of Th17, resulting in reduction of the level of proinflammatory cytokines including IL-17, IL-17F, IL-22 IL-1, IL-6, and IL-12 [71]. Vitamin C is another key vitamin that is indicated as an immunoregulatory supplement. Vitamin C is essential, cannot be synthesized in the human body and exerts both antioxidant and anti-inflammatory properties [192, 193]. Vitamin C has antioxidative properties, which arise from its ability to donate electrons, thus, protecting molecules from oxidative damage [71]. Vitamin C increases antiviral cytokines, like (IFN)-α/β, and controls the inflammatory responses in viral infections. Another immunoregulatory supplement is vitamin E, which is a potent antioxidant. Vitamin E triggers down-regulation of oxidative stress developed in Omicron infections by acting as a hydrogen donor to neutralize ROS.

Flavonoids are a group of naturally occurring polyphenols that are secondary metabolites in vegetables, grains, fruits, and tea. Besides their essential biological roles in plants, flavonoids show antioxidant, antimicrobial, anti-inflammatory, and other bioactivities in humans [77]. The bioactivity of flavonoids is acquired from their structural substitution patterns in their C6-C3-C6 rings. A group of 26 flavonoids like caflanone, hesperetin, and myricetin was tested for possible activity against Omicron (Table 1)**.**

Terpenoids are a modified class of terpenes, which are characterized by oxygen-containing hydrocarbons [83]. These naturally occurring lipids are derived from the five-carbon compound isoprene and are found as secondary metabolites in plants [83]. Terpenoids are bioactive against many diseases and are frequently used in pharmaceuticals. A group of five terpenoids (crocin, sarsasapogenin, ursonic acid, carvacrol, and glycyrrhizic acid) was tested for possible activity against Omicron (Table 1).

Alkaloids are a group of natural products that contain one or more nitrogen atoms (amido or amino) in their chemical structures [145]. Alkaloids are extracted from plant sources and are usually odourless and colourless crystalline solids in their pure forms [148]. There are more than 3000 known alkaloids, but this review focused on quinadoline B and scedapin C because they were tested for possible activity against Omicron (Table 1).

Glycosides are a large group of secondary metabolites that are derived from plant sources. Glycoside is a molecule within which a sugar portion is associated to a non-sugar moiety via a glycosidic bond, which may be phenol, alcohol, or sulphur compounds [159]. Four glycosides (DGCG, pelargonidin, and digitoxigenin) were tested for possible activity against Omicron (Table 1). Quinones are widespread biological pigments that are found in bacteria, fungi, higher plants, and even in animals. Quinones are fully conjugated cyclic dione compounds obtained from aromatic compounds such as benzene or naphthalene [161]. Anthraquinone was tested for possible activity against Omicron (Table 1). Monolignols are a group of natural products derived from phenylalanine via the phenylpropanoid pathway. Monolignols are monomeric units that build a lignin polymer in the cell walls of xylem, and sclerenchyma in plants [139]. Anethole and cinnamaldehyde are two examples of Monolignols that were tested for possible activity against Omicron (Table 1). Phenolic and polyphenolic compounds are naturally occurring compounds that are ubiquitously distributed in plants [165]. These compounds have many biological activities and have medicinal and industrial value. Phenolic compounds have a chemical structure composed of an aromatic ring with one or more hydroxyl substituents [165]. Ararobinol, gingerol, and other four compounds were tested for possible activity against Omicron (Table 1).

Carotenoids are a group of naturally occurring pigments of carotenes and their oxygenated derivatives (xanthophylls). Carotenoids are synthesized by photosynthetic organisms like plants, algae, cyanobacteria, fungi and non-photosynthetic bacteria [145]. Carotenoids are beneficial antioxidants that protect against free radicals and lower oxidative stress. Astaxanthin and polyketides emodin are two examples of carotenoids that were tested for possible activity against Omicron (Table 1). Miscellaneous natural compounds such as isochaetochromin D1, bisindigotin, edgeworoside C, adlumidine, asparagoside-C, asparagoside-D, and asparagoside-F have a potential effects against Omicron [145]. It is important to use these natural products in conjunction with chemical drugs to reach maximum antiviral effects, thus further study and trials are recommended.

## Role of natural products against Omicron virus

**Fig. 5:**
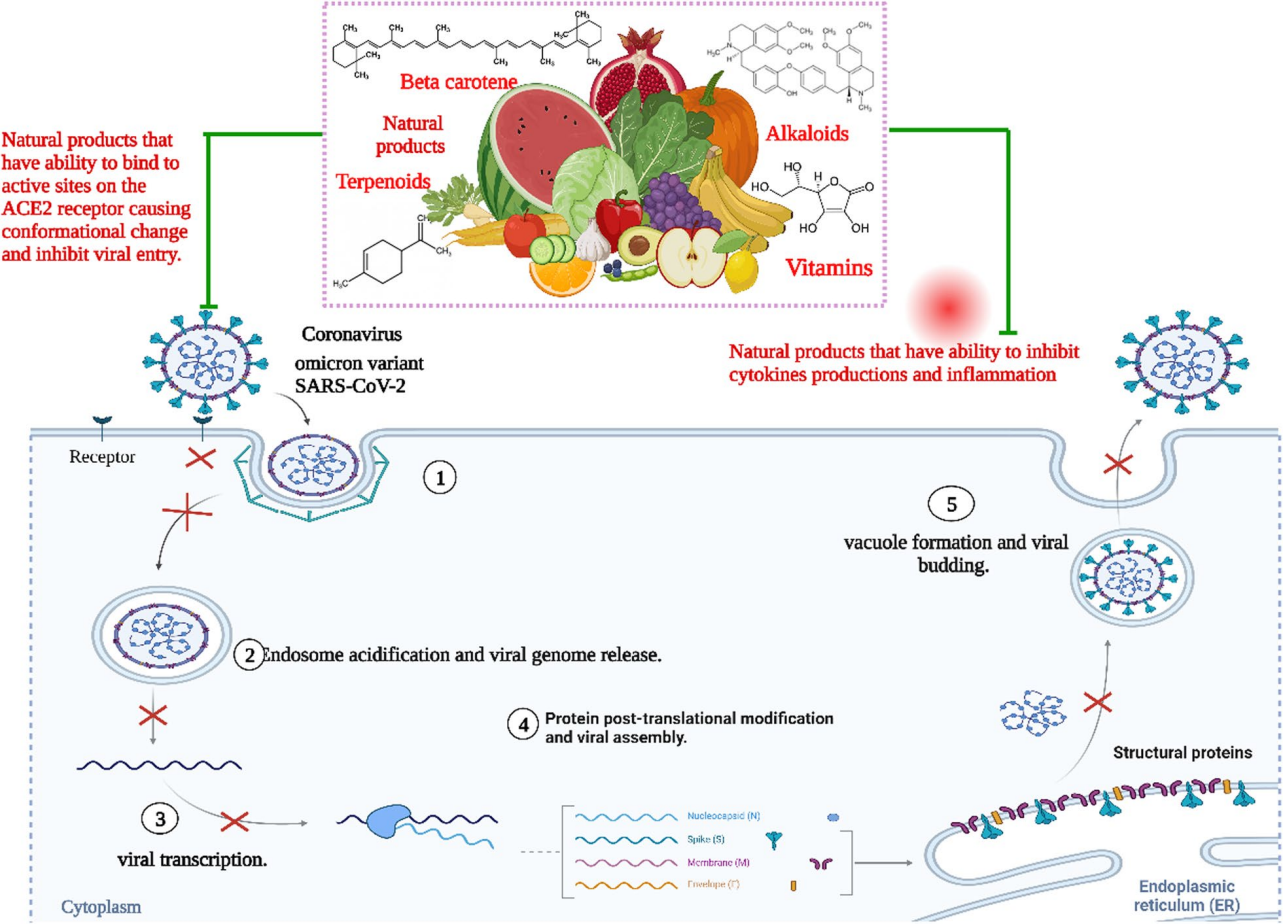
Natural products cause blocking of the ACE2 host cell, decrease inflammatory cytokines and inhibit omicron viral assembly

Natural products including flavonoids, terpenoids, alkaloids, glycosides, miscellaneous compounds, carotenoids, phenolic and polyphenolic compounds were overlapped with the ACE-2 interface and disrupted the interaction of ACE-2 with RBD of Omicron, as shown in Fig. 4 [18]. Natural products not only disturb the binding interaction of the virus, but also reduces pulmonary complications related to Omicron infection by limiting thromboembolism [19]. Thus, natural products show promise against the Omicron infection. COVID-19 is a positive-strand virus, which injects its + ssRNA into the host cell cytoplasm, which rapidly translates into the two replicase polyproteins pp1a and pp1ab [194]. Wu et al. demonstrated that the protease 3Clpro or Mpro is essential for the processing of the replicases [18, 146]. Mpro is targeted by a wide range of naturally produced antiviral drugs [195, 196], which inhibit viral replication, as shown in Fig. 5. Natural products are supposed to control the severity of Omicron because of their role in respiratory and cardiovascular health maintenance [20]. Clinical trials reported the beneficial effect of dietary hesperidin and other natural products in animals suffering neurodegenerative disorders and febrile seizures [73, 197–199]. Flavonoids control inflammatory response gene expression, and genes involved in atherogenesis, and cytoskeletal organization [200]. Pro-inflammatory cytokines such as interleukins are released upon viral infection, which may progress to SARS [201]. Flavonoids control the production of inflammatory cytokines in microglia, and reduce the formation of ROS induced by lipopolysaccharides [202]. Omicron, during host cell infection, cause breaks in the oxidative balance inducing oxidative stress. Oxidative stress enables the virus to continue its life cycle and finally, host cell death occurs [94]. Severe omicron infection causes disruption of iron metabolism resulting in the release of free haeme and hyperferritinaemia [203, 204] besides the production of ROS, which results in oxidative stress. Natural products including ascorbic acid (vitamin C) and hesperidin are powerful antioxidants that target many ROS like superoxide, hydroxyl radicals and nitric oxide production [74, 78]. Thus, it is important to focus on natural products as antivirals due to their beneficial effects against viral infection of vaccinated and unvaccinated people with few side effects, as shown in Fig. 4.

## Results of In vitro docking

The spike glycoprotein of the Omicron variant binds cellular receptors of the angiotensin-converting enzyme 2 (ACE2) in the respiratory tract and lungs to facilitate infection and ultimately entry into cells [77, 205–207]. Chloroquine inhibits CoV-2 activity, which prompted the investigation of possible chloroquine binding to ACE2 [208]. Molecular docking studies are conducted to examine the *in-silico* inhibition effect of inhibitors towards the ACE2 metallopeptidase domain (PDB ID: 1R4L) compared with chloroquine. Plausible binding modes with target binding sites and their interactions with protein hot spots (key amino acids) are investigated.

**Fig. 6:**
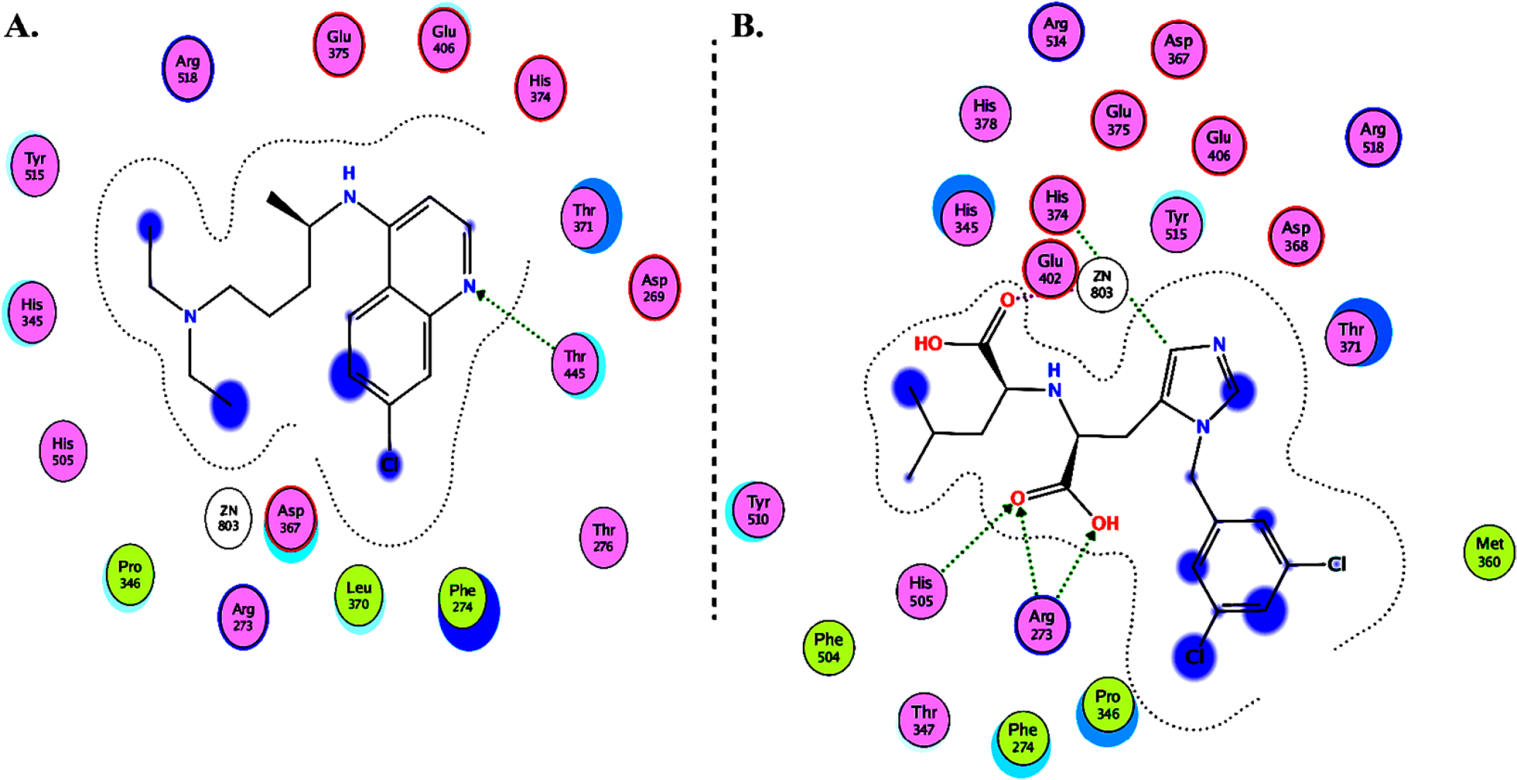
2D diagrams of **A** Chloroquine and **B** XX5 ligand showing their interaction with the ACE2 metallopeptidase domain active site

Molecular Operating Environment (MOE 2010) software is used to perform docking studies [209] according to previously described procedures [210]. All structural minimizations were performed until an RMSD gradient of 0.05 kcal∙mol^−1^ Å^−1^ with *MMFF94x* force field and partial charges were automatically calculated. X-ray crystallographic structure of the target protein was obtained from a protein databank [211]. All water molecules were removed, and the target protein was then prepared for docking using *Protonate 3D* protocol in MOE with default parameters. The co-crystalized ligand, as shown in Fig. 6, was used to define the binding site for docking simulation. *Triangle Matcher placement* method and *London dG* scoring function were used for docking and scoring. The docking protocol was first validated by self-docking of the co-crystallized ligand near the binding site of the protein. Then, the validated docking protocol (RMSD < 2) was utilized to study the ligand-receptor interactions at the protein binding site for the inhibitors to predict their binding mode and binding affinity.

Table 2 summarizes the docking results of the groups including flavonoids, terpenoids, alkaloids, glycosides, carotenoids, monolignols, phenolic and polyphenolic compounds, and miscellaneous compounds. The results indicate that most of the compounds (except for monolignols) could bind with high affinity to the protease sites on the ACE2 receptor causing conformational change to inhibit viral entry of Omicron. In addition, delphinidin 3,3′-di-glucoside-5-(6-P-coumarylglucoside) (DGCG) could bind to the ACE2 receptor (with binding energy S = − 34.03 kcal/mol) three times more effective than the standard chloroquine (S = − 10.33 kcal/mol).

**Table 2:** Docking energy scores and amino acids involved in binding for XX5 ligand, chloroquine, and the reported inhibitors docked with the ACE2 receptor

Name	Compound	Docking score (kcal/mol)
Cocrystalized ligand (XX5 ligand)	− 16.82	Arg273, His374, Glu402, His505, and Zn^2+^ ion
Chloroquine (Standard)	− 10.33	Thr445
*Flavonoids*		
Caflanone	− 15.35	His374, Thr445, and Pro346
Hesperetin	− 15.63	Thr347 and Zn^2+^ ion
Myricetin	− 18.02	Glu406 and Pro346
Pectolinarin	− 22.56	Arg518, His378, His374, Glu402, His505, and Zn^2+^ ion
Quercetin	− 16.51	Glu406 and Pro346
Baicalin	− 18.64	His374, Glu375, and Zn^2+^ ion
Diosgenin	− 16.20	Tyr515, Asp367, and Zn^2+^ ion
Baicalein	− 14.94	Arg273
Luteolin	− 16.73	Glu375 and Asp367
Cyanidin	− 16.36	Asp367
Kaempferol	− 14.87	Arg273 and Thr445
Rutin	− 24.22	Arg518, Glu375, Tyr515, His345, His374, Glu402, and Zn^2+^ ion
Narcissoside	− 21.40	Asp269, Asn149, Thr371, Glu375, His505, Tyr515, Met270, His345, His374, Glu402, and Zn^2+^ ion
Isorhamnetin-3-O-B-D-Glucoside (IRG)	− 17.39	His374 and Thr371
Calendoflaside	− 21.42	Asn149, Asp368, and Glu406
Procyanidin	− 21.75	Asp367 and Pro346
Nicotiflorin	− 20.91	Pro346, Arg518, Ala348, His345, and His505
Broussochalcone A	− 17.94	Pro346
Broussoflavan A	− 16.96	Asp368, Phe274, Glu375, and Zn^2+^ ion
Fisetin	− 14.49	Glu406
Licoisoflavone B	− 15.90	Thr347, Glu375, and Zn^2+^ ion
Isolicoflavonol	− 16.65	Zn^2+^ ion
Limonene	− 6.94	Tyr510
b-Carophyllene	− 8.977	Zn^2+^ ion
Ascorbic acid	− 12.55	His345 and Zn^2+^ ion
*Terpenoids*		
Crocin	− 23.85	Glu406, Leu120, Thr445, Gln442, Lys441, and Trp349
Sarsasapogenin	− 15.16	Asp367, Tyr515, and Zn^2+^ ion
Ursonic Acid	− 13.44	Arg273 and His374
Carvacrol	− 10.19	Glu375
*Alkaloids*		
Quinadoline B	− 13.50	His374
Scedapin C	− 15.68	Thr371
Berberine	− 16.52	His378 and Ala348
Nigellidine	− 11.73	Glu402
Noscapine	− 15.35	Arg518
Oxoturkiyenine	− 13.97	Arg518
3α,17α-Cinchophylline	− 15.22	Arg273
Speciophylline	− 13.47	Arg518, Phe274, and Zn^2+^ ion
Cadambine	− 20.04	Arg518, Glu375, Tyr510, His505, His345, and Zn^2+^ ion
*Glycosides*		
Delphinidin 3,3′-Di-Glucoside-5-(6-P-Coumarylglucoside) (DGCG)	− 34.03	Glu406, Phe274, His345, Tyr127, Asn149, Lys363, Asp367, and Asp368
Pelargonidin	− 15.47	Asp367
Digitoxigenin	− 14.35	Glu375, Thr445, Phe274, and Zn^2+^ ion
*Carotenoids*		
Astaxanthin	− 17.48	Ser47 and Gln442
Emodin	− 14.08	Arg518
*Monolignols*		
Anethole	− 8.967	Thr347
Cinnamaldehyde	− 9.208	Zn^2+^ ion
*Phenolic and polyphenolic compounds*		
Curcumin	− 13.5	Asp269, His345, and Zn^2+^ ion
Gallocatechin gallate	− 21.94	Glu375, Asp367, and Asp368
Ararobinol	− 16.00	Asp368 and Tyr127
Gingerol	− 12.86	Glu375 and His345
*Miscellaneous compounds*		
Isochaetochromin D1	− 20.96	Asn149, Asn277, Thr371, and Asp368
Bisindigotin	− 14.66	Glu406 and Phe274
Edgeworoside C	− 17.07	Glu375, Tyr515, Phe274, His345, and Zn^2+^ ion
Adlumidine	− 12.79	Thr371
Asparagoside-C	− 21.66	Glu406, Tyr127, Arg518, and Thr519
Asparagoside-D	− 24.66	Thr445, Thr276, His345, Asp367, Asp368, and Arg518
Asparagoside-F	− 29.62	Arg518, Thr445, Asp367, Thr371, and Zn^2+^ ion
3-(3-Methylbut-2-enyl)-3,4,7-trihydroxyflavane (MTHF)	− 14.87	Glu375 and Zn^2+^ ion
Kazinol F	− 17.11	Pro346
Kazinol J	− 18.08	Asp368, Thr371, and His374
Cinnamyl acetate	− 8.858	Arg518, Glu375, and Zn^2+^ ion
L-4-terpineol	− 9.345	Zn^2+^ ion
Allicin	− 8.180	Glu402, His505, His345, and Zn^2+^ ion
Anthraquinone	− 9.508	Arg518
Gallocatechin gallate	− 21.94	Glu375, Asp367, and Asp368
Ararobinol	− 16.00	Asp368 and Tyr127
Gingerol	− 12.86	Glu375 and His345
*Miscellaneous compounds*		
Isochaetochromin D1	− 20.96	Asn149, Asn277, Thr371, and Asp368
Bisindigotin	− 14.66	Glu406 and Phe274
Edgeworoside C	− 17.07	Glu375, Tyr515, Phe274, His345, and Zn^2+^ ion
Adlumidine	− 12.79	Thr371
Asparagoside-C	− 21.66	Glu406, Tyr127, Arg518, and Thr519
Asparagoside-D	− 24.66	Thr445, Thr276, His345, Asp367, Asp368, and Arg518
Asparagoside-F	− 29.62	Arg518, Thr445, Asp367, Thr371, and Zn^2+^ ion
3-(3-Methylbut-2-enyl)-3,4,7-trihydroxyflavane (MTHF)	− 14.87	Glu375 and Zn^2+^ ion
Kazinol F	− 17.11	Pro346
Kazinol J	− 18.08	Asp368, Thr371, and His374
Cinnamyl acetate	− 8.858	Arg518, Glu375, and Zn^2+^ ion
L-4-terpineol	− 9.345	Zn^2+^ ion
Allicin	− 8.180	Glu402, His505, His345, and Zn^2+^ ion
Anthraquinone	− 9.508	Arg518

**Fig. 7:**
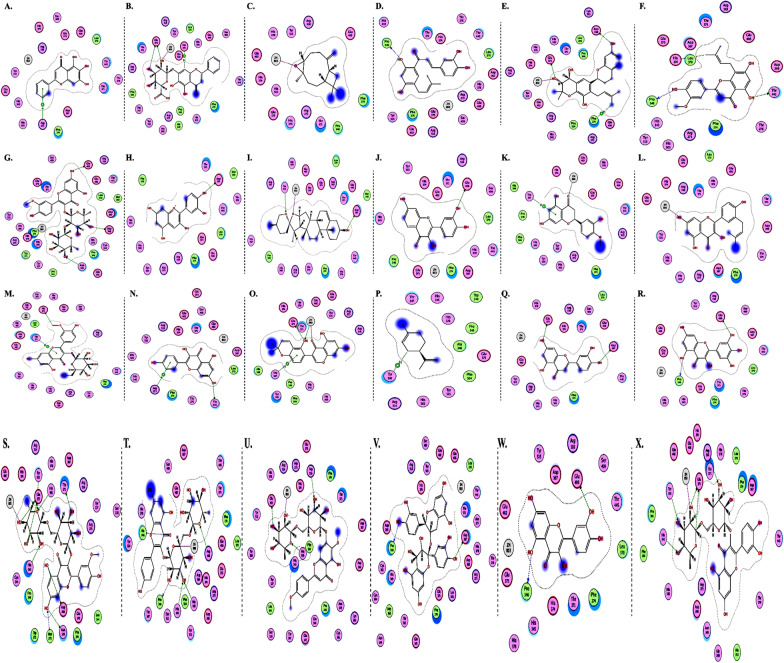
2D diagrams of **A** Baicalein, **B** Baicalin, **C** Carophyllene, **D** Broussochalcone A, **E** Broussoflavan A, **F** Caflanone, **G** Calendoflaside, **H** Cyanidin, **I** Diosgenin, **J** Fisetin, **K** Hesperetin, **L** Isolicoflavonol, **M** 1_Isorhamnetin-3-O-B-D-Glucoside (IRG), **N** Kaempferol, **O** Licoisoflavone B, **P** Limonene, **Q** Luteolin, **R** Myricetin, **S** Narcissoside, **T** Nicotiflorin, **U** Pectolinarin, **V** Procyanidin, **W** Quercetin and **X** Rutin showing their interactions with the ACE2 metallopeptidase domain active site

**Fig. 8:**
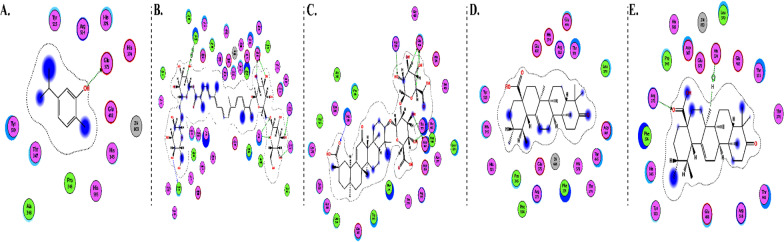
2D diagrams of **A** Carvacrol, **B** Crocin, **C** Glycyrrhizic Acid, **D** Sarsasapogenin and **E** Ursonic Acid, showing their interactions with the ACE2 metallopeptidase domain active site

**Fig. 9:**
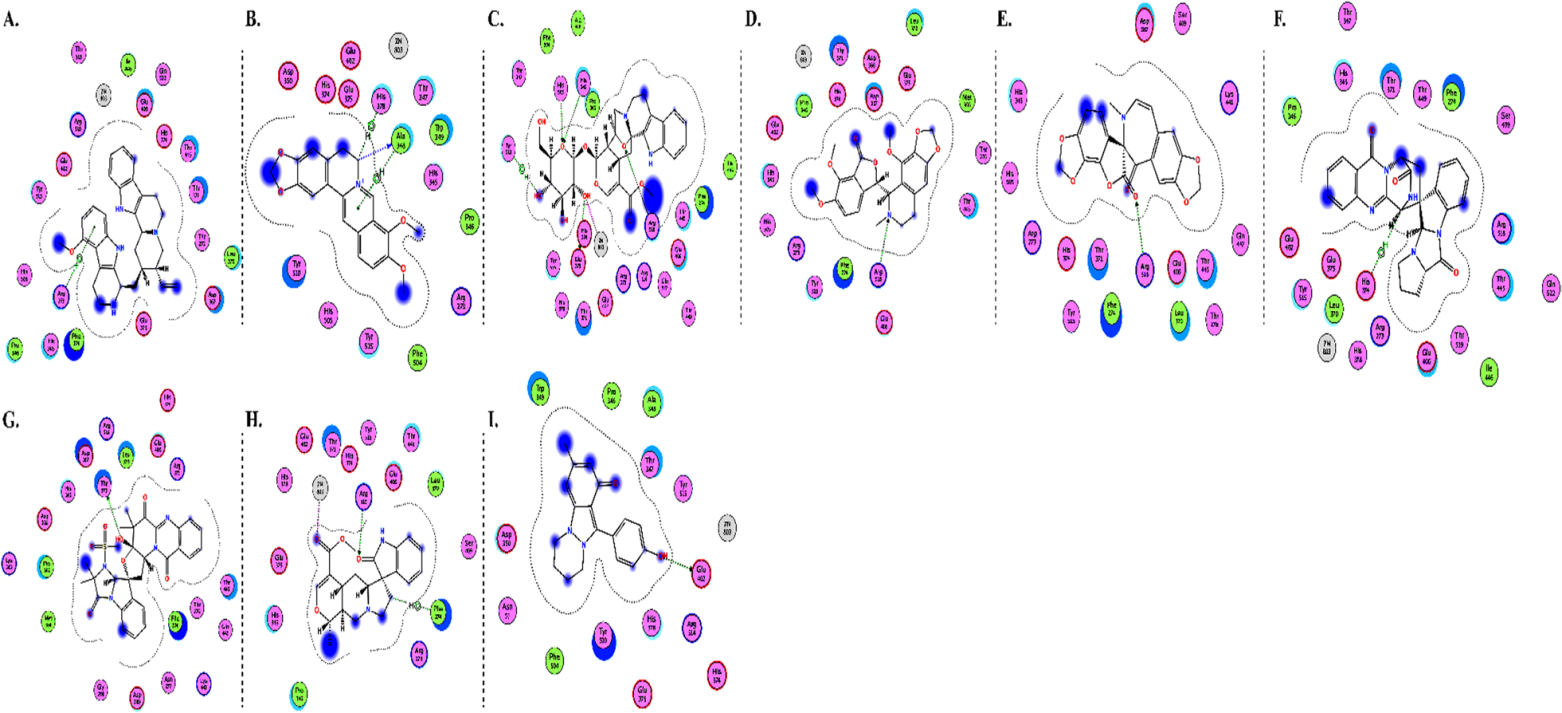
2D diagrams of **A** 3a,17a-Cinchophylline, **B** Berberine, **C** Cadambine, **D** Noscapine, **E** Oxoturkiyenine, **F** Quinadoline B, **G** Scedapin C, **H** Speciophylline and **I** Nigellidine, showing their interactions with the ACE2 metallopeptidase domain active site

**Fig. 10:**
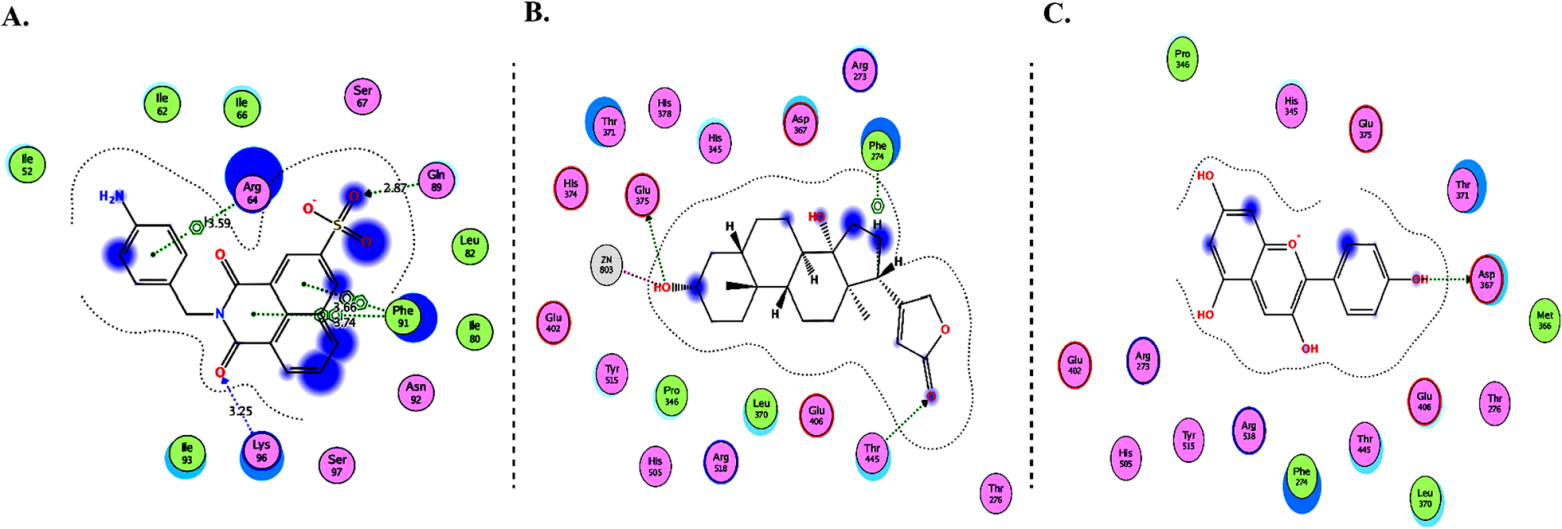
2D diagrams of **A** Delphinidin 3,3-Di-Glucoside-5-(6-P-Coumarylglucoside) (DGCG), **B** Digitoxigenin and **C** Pelargonidin, showing their interactions with the ACE2 metallopeptidase domain active site

**Fig. 11:**
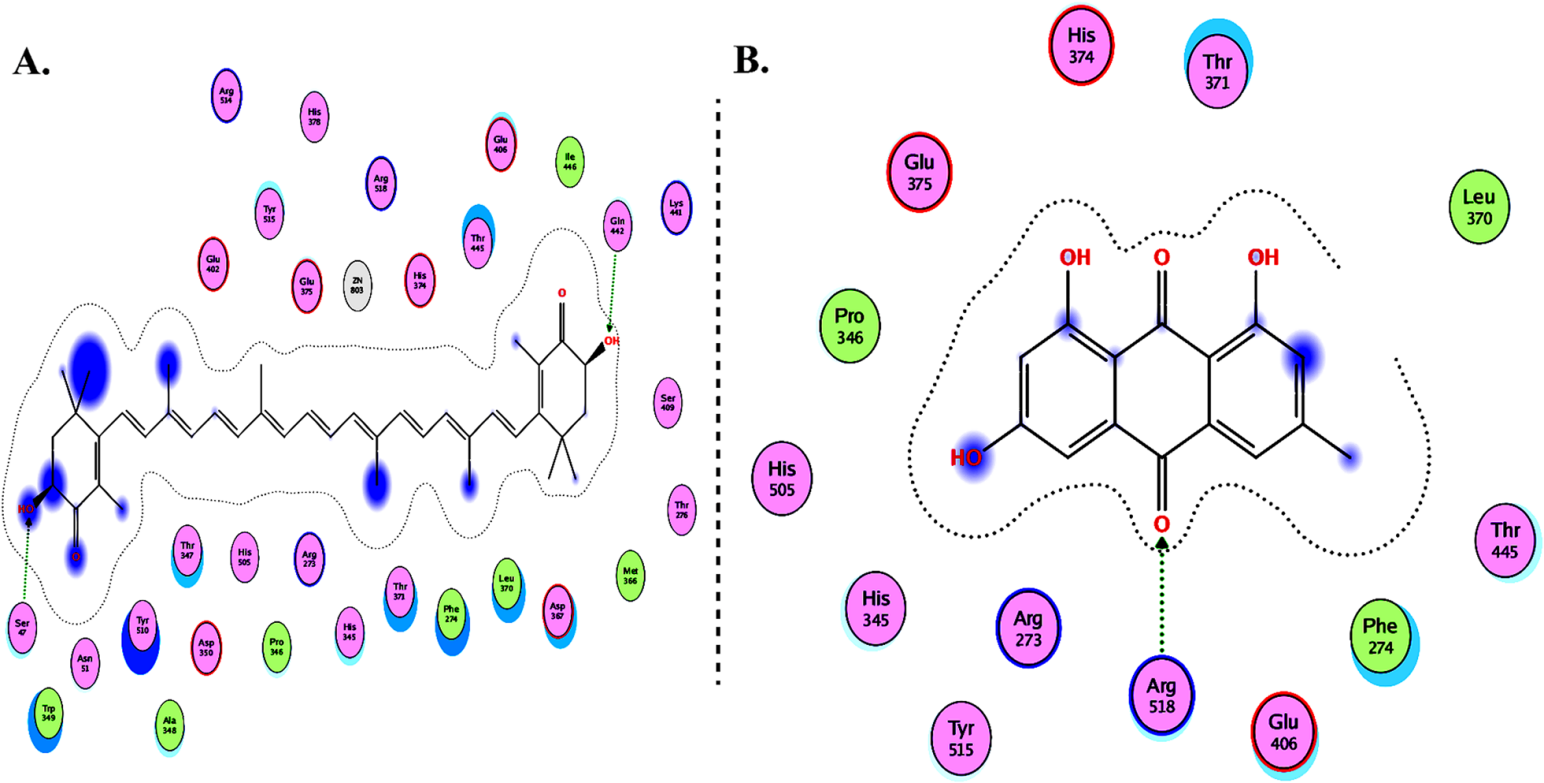
2D diagrams of **A** Astaxanthin and **B** Emodin, showing their interactions with the ACE2 metallopeptidase domain active site

**Fig. 12:**
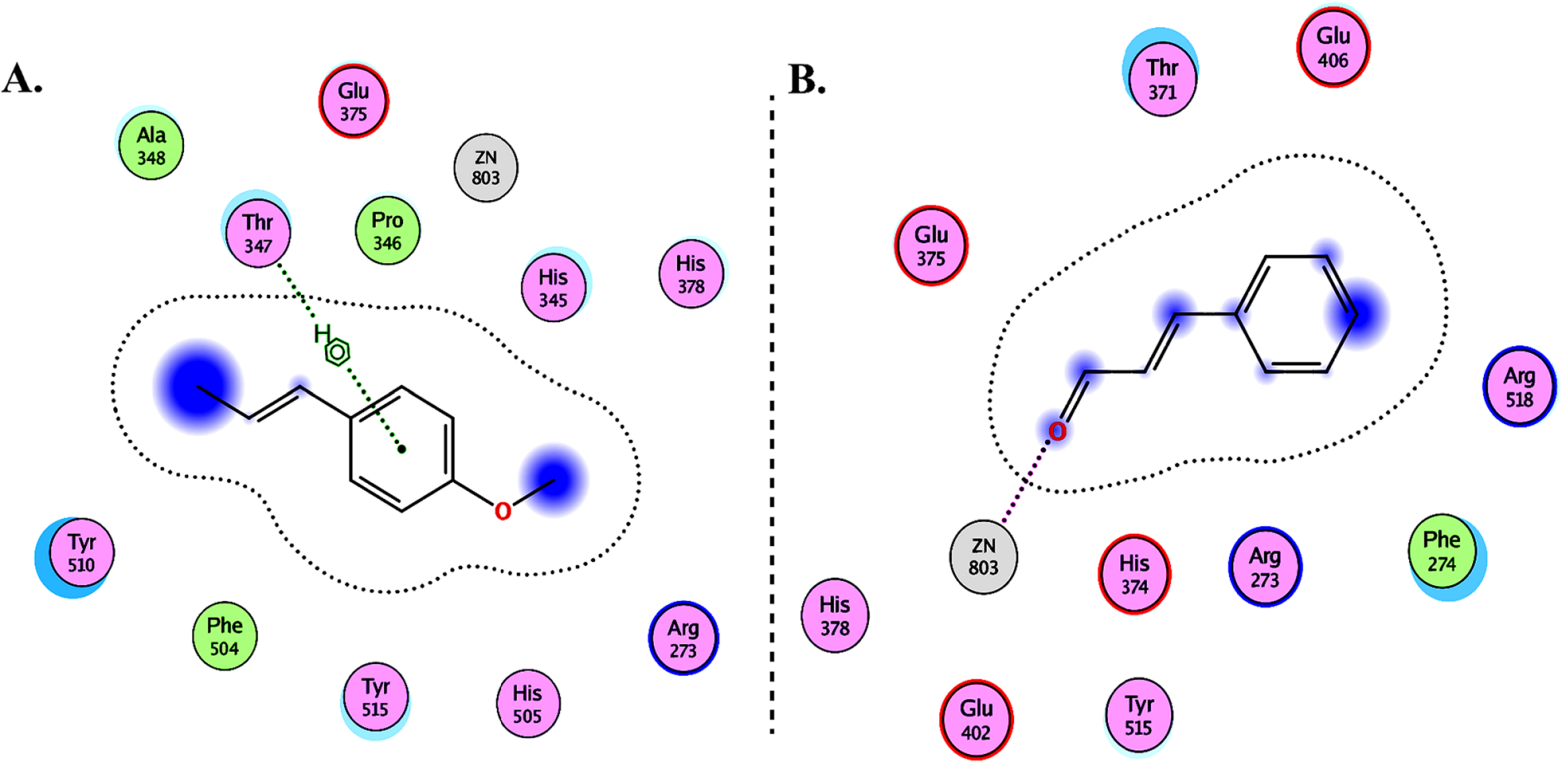
2D diagrams of **A** Anethole and **B** Cinnamaldehyde, showing their interactions with the ACE2 metallopeptidase domain active site

**Fig. 13:**
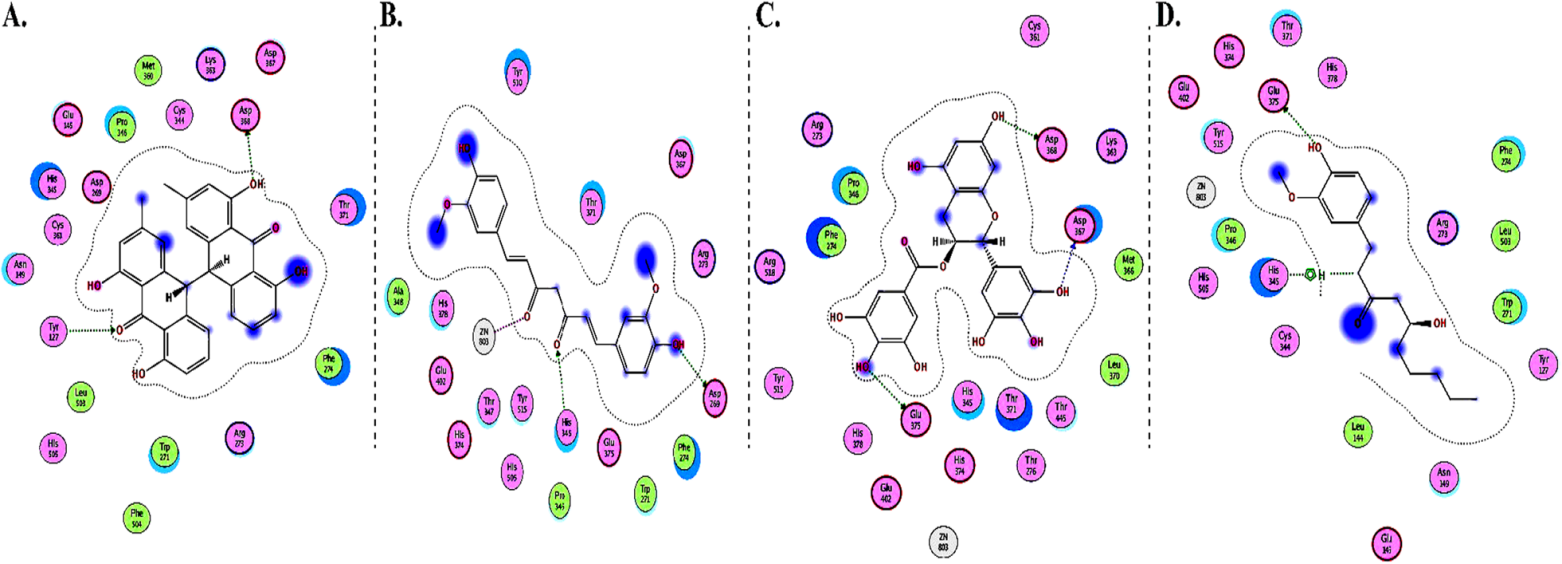
2D diagrams of **A** Ararobinol, **B** Curcumin, **C** Gallocatechin gallate and **D** Gingerol, showing their interactions with the ACE2 metallopeptidase domain active site

**Fig. 14:**
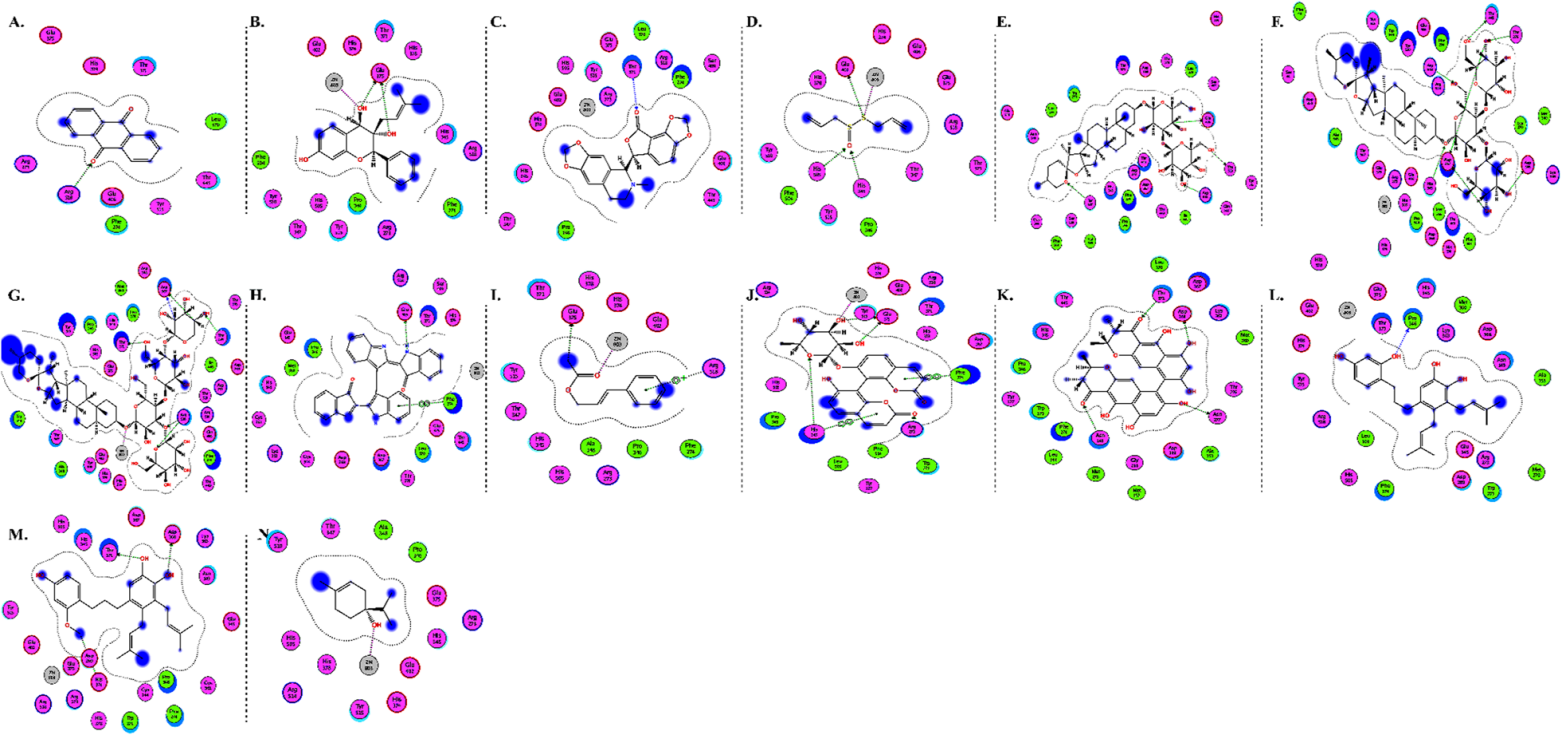
2D diagrams of **A** Anthraquinone, **B** 3-(3-Methylbut-2-enyl)-3,4,7-trihydroxyflavane (MTHF), **C** Adlumidine, **D** Allicin, **E** Asparagoside-C, **F** Asparagoside-D, **G** Asparagoside-F, **H** Bisindigotin, **I** Cinnamyl acetate, **J** Edgeworoside C, **K** Isochaetochromin D1, **L** Kazinol F, **M** Kazinol J and **N** L-4-terpineol, showing their interactions with the ACE2 metallopeptidase domain active site

The interactions of DGCG and chloroquine with the ACE2 metallopeptidase domain active site displays a single hydrogen bond with the residue of Thr445 for chloroquine. Nine hydrogen bond interactions for DGCG are found with Glu406, Phe274, His345, Tyr127, Asn149, Lys363, Asp367, and Asp368 in addition to a pi–pi interaction with Phe274. The interactions of the other compounds with the ACE2 metallopeptidase domain active site are shown in Figs. 7, 8, 9, 10, 11, 12, 13 and 14.

## Discussion

The continuous genetic modifications of the SARS-CoV-2 virus through DNA mutations in its spike protein result in the emergence of novel variants such as Alpha, Beta, Delta, and Omicron. The Omicron variant stands out with its remarkably high replication rate, multiplying approximately 70 times faster than other variants, albeit displaying reduced severity [2]. Despite extensive vaccination efforts, the prevalence of Omicron infections persists, suggesting that a reliance solely on vaccinations may not be sufficient to eliminate the pandemic. The World Health Organization (WHO) underscores that Omicron can infect both vaccinated and unvaccinated individuals due to mutations in the spike protein, enabling evasion of the immune response and vaccines. Notably, Omicron's propensity for spreading is more pronounced among the unvaccinated, which potentially contributes to the emergence of novel strains. The Omicron variant exhibits a significant number of mutations, exceeding 60 substitutions, deletions, and insertions, including around 30 alterations in the spike protein [3]. These mutations confer heightened transmissibility within a limited timeframe, driven by their rapid dissemination and ability to evade the body's immune defenses, even among individuals who have received dual vaccination [8]. The vaccine remains the primary safeguard against Omicron; however, its effectiveness, feasibility, and safety are yet to be definitively established. Ongoing clinical trials are evaluating these aspects, while numerous drugs are still undergoing testing to combat Omicron infection.

Recent research highlights the potential of dietary components rich in polyphenols and vitamins (A, C, D, E) to mitigate the transmission and severity of infectious diseases [10, 12]. For instance, the work of Wallace et al. underscores that food-derived polyphenols enhance human immunity against oxidative stress, demonstrating anti-inflammatory, antiviral, and antibacterial properties, as well as potential benefits in preventing cardiovascular disease, atherosclerosis, and cancer [12]. In a similar vein, Wu et al. [18] report that natural compounds, including flavonoids, terpenoids, alkaloids, glycosides, carotenoids, and phenolic and polyphenolic compounds, interact with the ACE-2 interface, leading to the disruption of ACE-2 interaction with the receptor-binding domain (RBD) of SARS-CoV-2. Furthermore, the role of citrus flavanones in cardiovascular protection is elucidated by the findings of Chanet and colleagues [20]. Natural products assume a potentially pivotal role as antivirals, given their capacity to maintain cardiovascular and respiratory health, thereby potentially reducing the susceptibility of Omicron-infected individuals to severe complications [20]. Abubakar et al. [212] provide evidence that natural products can modulate the angiotensin-converting enzyme 2 (ACE2), holding promise as potential therapies for COVID-19 [213]. Their study indicates the potential for medicinal properties to mitigate viral invasion by directly or indirectly modulating ACE2 activity, thus ameliorating COVID-19. Notably, ethnomedicinal plants containing bioactive compounds that can modulate ACE2-associated events are highlighted as potential interventions to prevent and mitigate SARS-CoV-2 fusion and entry [213]. Additionally, the work of Wasilewicz and collaborators [214] underscores the inhibition of SARS-CoV-2 infection through natural products targeting viral proteases [215]. Importantly, these natural products exhibit inhibitory effects against both the main protease (Mpro) and papain-like protease (PLpro) of SARS-CoV-2 [215].

In light of this context, a comprehensive array of natural compounds, encompassing flavonoids, terpenoids, alkaloids, glycosides, carotenoids, monolignols, phenolic and polyphenolic compounds, and other miscellaneous compounds, emerges as potential natural interventions against the coronavirus family, including the Omicron variant. Our investigation involved molecular docking studies to assess the in-silico inhibitory effects of natural inhibitors on the ACE2 metallopeptidase domain (PDB ID: 1R4L), juxtaposed with chloroquine. The outcomes of our docking simulations reveal that the majority of these compounds (except monolignols) exhibit robust binding affinity to the protease sites on the ACE2 receptor, inducing a conformational change that inhibits viral entry of the Omicron variant. The interactions of these compounds with the active site of the ACE2 metallopeptidase domain are visualized in Figs. 7, 8, 9, 10, 11, 12, 13 and 14 and Table 2. Particularly noteworthy is the observation that delphinidin 3,3′-di-glucoside-5-(6-P-coumarylglucoside) (DGCG) displays a binding energy (S) of − 34.03 kcal/mol upon binding to the ACE2 receptor, signifying an approximately three-fold greater potency compared to the standard chloroquine (S = − 10.33 kcal/mol). DGCG's interaction involves a singular hydrogen bond with the Thr445 residue for chloroquine, while it forms nine hydrogen bonds with Glu406, Phe274, His345, Tyr127, Asn149, Lys363, Asp367, and Asp368, in addition to a pi–pi interaction with Phe274. The docking interaction of DGCG with the ACE2 metallopeptidase domain holds the potential to obstruct viral entry into the host [213]. These findings align harmoniously with a study by Al-Shuhaib and co-workers, further substantiating DGCG's viability as a candidate natural compound for suppressing ACE2-virus interaction [215]. This investigation attributes DGCG's antiviral activity to its favorable pharmacokinetics, drug likeness, and toxicity profile, encompassing high gastrointestinal absorption, intracellular metabolism, excretion, and minimal toxicity. Notably, DGCG is demonstrated to hinder virus adsorption to cells and impede virus release from infected cells.

ACE2, situated on cell surfaces, plays a pivotal role in blood pressure regulation and the renin–angiotensin–aldosterone system, serving as the cellular entry point for SARS-CoV-2. DGCG's binding to the ACE2-binding domain of the virus bears the potential to hinder or attenuate ACE2-virus interaction, thereby potentially inhibiting viral entry. Moreover, DGCG could exert influence on ACE2 expression at transcriptional or translational levels, indirectly affecting the receptor's availability for viral binding. Additionally, DGCG's capacity to modulate intracellular signaling pathways might indirectly impact ACE2 activity. Lastly, DGCG could prompt conformational alterations in the ACE2 receptor or the virus's spike protein, thereby potentially impeding their effective interaction [216–221].

The present study showcases the substantial therapeutic potential of natural products through their robust inhibitory effects, as evidenced by our in-silico docking investigations, in contrast to the standard drug chloroquine. This review comprehensively explores various categories of natural products with the capability to inhibit viral infection, thereby offering potential benefits to both vaccinated and unvaccinated individuals, while also minimizing adverse effects. These natural products hold promise as adjuvants in combination with antiviral drugs, thereby augmenting antiviral efficacy against the Omicron variant. The findings presented here underscore the significance of natural interventions in combatting the transmission of B.1.1.529 Omicron and encourage further exploration of these natural compounds as a strategic approach to address.

## Conclusion and future perspectives

Due to high transmissibility and effective ACE2‐mediated infection of the Omicron variant, which is the most serious new variant of SARS-cov-2 These characteristics result from 30 mutations in the Omicron spike that enable Omicron to evade the immune response. The WHO scientists are working to find drugs and improve vaccines to inhibit the spread of Omicron. Inclusion of foods and vitamins as therapeutic supplements are also studied. The therapeutic effect of natural products is very high compared to the standard drug chloroquine. In this review, we performed molecular docking simulations revealed that most of the natural compounds could bind with high affinity to the ACE2 metallopeptidase domain active site. And we observed that DGCG is the most effective natural product against omicron compared to chloroquine.

Unfortunately, natural products have drawbacks such as lack of specificity and selectivity in host cells. Thus, it is important to conduct clinical trials on natural products to ensure their safety and effectiveness against the virus.

## Data Availability

The data that support the findings of this study are available from the corresponding author upon reasonable request.
